# Artificial intelligence in spatial transcriptomics: a review of methods, datasets, and directions

**DOI:** 10.1093/bib/bbag569

**Published:** 2026-10-18

**Authors:** Neelam Sharma, Rabia Şen, Cemal Erdem

**Affiliations:** Department of Medical Biosciences, Umeå University, 901 85, Umeå, Sweden; Science for Life Laboratory (SciLifeLab), Umeå University, 901 85, Umeå, Sweden; Department of Medical Biosciences, Umeå University, 901 85, Umeå, Sweden; Science for Life Laboratory (SciLifeLab), Umeå University, 901 85, Umeå, Sweden; Department of Medical Biosciences, Umeå University, 901 85, Umeå, Sweden; Science for Life Laboratory (SciLifeLab), Umeå University, 901 85, Umeå, Sweden

**Keywords:** spatial transcriptomics, artificial intelligence, large language models, deep learning, omics analysis, multi-omics integration

## Abstract

Spatial transcriptomics (STs) measures gene expression while retaining the location of transcripts or cells within tissue sections. Unlike bulk RNA sequencing, which averages expression across cell populations, and single-cell RNA sequencing, which generally requires tissue dissociation, ST links molecular profiles to tissue architecture. This spatial information supports the analysis of tissue heterogeneity, cellular organization, and potential intercellular interactions. The spatially resolved nature of ST has unlocked diverse applications in fields such as cancer, neuroscience, reproductive biology, and plant biology. In parallel, the development of publicly accessible databases and repositories has facilitated large-scale, multi-cohort comparisons and benchmarking. The rapid expansion and increasing complexity of these datasets have driven the adoption of machine learning (ML), deep learning (DL), and artificial intelligence (AI) techniques. These computational tools support a range of tasks, including data pre-processing, image segmentation, spatial domain identification, and pattern recognition. Moreover, they are instrumental in integrative multi-omics analyses and predictive modeling. This AI-focused review provides necessary background on ST technologies and databases and evaluates the use of statistical, ML, and DL methods across the analytical workflow, from image and sequencing-data preprocessing to spatial analysis and LLM-assisted interpretation. We compile key resources related to datasets, AI-driven analytical frameworks, and ST data analysis pipelines, and discuss current challenges, limitations, and future directions for AI-based tools in spatial omics.

## Introduction

Multicellular organisms are made up of trillions of cells, each functioning as an essential building block of life. These diverse cells are organized in a specific pattern to constitute tissues and organs, and they perform specialized biological functions. Despite sharing the same genome, these cells differ significantly in function, structure, and behavior, and exhibit distinct gene expression patterns. This variation is caused not only by their tissue type but also by the surrounding microenvironment. Therefore, understanding the distinctive patterns of gene expression and spatial arrangement of cells within those tissues is essential for studying tissue function and the underlying molecular mechanisms of diseases [1].

The emergence of next-generation sequencing (NGS) has changed our ability to analyze gene expression at a genome-wide level [2]. It has transformed and opened new avenues for understanding the complex traits of biological research by enabling high-throughput and cost-effective sequencing of whole transcriptomes [3]. Bulk RNA sequencing and single-cell RNA sequencing (scRNA-seq) are powerful applications of NGS [4, 5]. Bulk RNA-seq quantifies RNA transcripts from tissues, averaging gene expression profiles across the entire cell population and thereby losing cellular heterogeneity. Single-cell RNA-seq enables profiling at the individual cell level while retaining the cellular heterogeneity but disrupting the original tissue architecture, resulting in loss of cellular spatial information, which is essential for understanding cell–cell interactions and tissue functionality [6–8]. Therefore, retaining spatial information is necessary for a comprehensive understanding of tissue architecture and cellular interactions within.

To circumvent the limitations of conventional single-cell technologies, spatial transcriptomics (STs) has emerged as an innovative approach that captures both gene expression profiles and spatial context across tissues [9, 10]. It generates high-resolution maps of gene expression within their native spatial context of intact tissue samples, revealing how cellular identity and function are shaped by their spatial environment [11, 12]. ST assigns each cell a unique spatial label that contains 2D spatial coordinates (x, y positions within a tissue section), and in some 3D implementations, volumetric coordinates (x, y, z), enabling accurate positioning of each cell sub-population within or across the original tissue sections [13, 14]. By providing insights into tissue architecture, ST has elucidated our understanding of tissue function, cellular interaction patterns, and microenvironment-specific gene regulation [15–17].

STs complements and competes with several other approaches for characterizing cellular identity and tissue organization. Apart from bulk and single-cell RNA-seq, multiplexed immunofluorescence (e.g. CODEX/PhenoCycler [18], t-CyCIF [19]) preserves spatial information and can profile 40–60 proteins simultaneously but is limited to protein-level readouts and pre-selected antibody panels. Live-cell imaging combined with single-molecule tracking can capture dynamic RNA processes in real time but is restricted to cultured cells or thin tissue sections. Among these, ST uniquely combines unbiased transcriptome-wide gene expression with spatial context in intact tissues, providing a molecular resolution of cellular heterogeneity and organization that none of the competing methods can achieve alone. However, ST currently lags behind multiplexed imaging in protein-level throughput, live-cell capture, and, for sequencing-based methods, spatial resolution. Integrative multi-modal approaches that combine ST with proteomics, epigenomics, or live imaging are therefore an active area of development, discussed throughout this review.

ST can tackle many biological questions related to single-cell technology and other vital issues that single-cell technology cannot resolve alone. It has a wide range of applications across various fields, including cancer, neuroscience, developmental biology, immunology, mouse biology, and plant biology. In cancer research, ST improves the analysis of tumor transcriptomic signatures and their associated cells, allowing for the profiling of spatial heterogeneity within the tumor microenvironment (TME) and providing deeper insights into tumor progression [20–23]. In neuroscience, ST facilitates the study of the spatial progression of neurodegenerative diseases, such as Alzheimer’s, amyotrophic lateral sclerosis (ALS), and multiple sclerosis [24–26]. In plant biology, ST offers insights into plant microbiota interactions, metabolic pathway analysis, stress responses, and crop improvement [27–29]. ST technologies have significant potential for clinical and translational applications across various disease fields.

ST technologies, employing various approaches, have resulted in the generation of vast amounts of data, including spatial and gene expression information. The rapid accumulation of these datasets has led to the development of various publicly accessible databases and well-curated repositories. These easily accessible resources facilitate comparative studies and large-scale computational analyses. The rapid expansion and increasing complexity of these datasets have driven the adoption of machine learning (ML), deep learning (DL), and artificial intelligence (AI) techniques to analyze and interpret such complex spatial data. Importantly, throughout this review, we use the term *AI* broadly to encompass the hierarchical family of methods including ML, DL, and generative/foundation models. Where methods are discussed at a specific level of this hierarchy, we use the more precise term (ML, DL, or LLM) accordingly. AI- and ML-based methods are widely used for image segmentation, spatial domain identification, and linking histological images with spatial gene expression. They also enable integration of ST data with other omics modalities such as proteomics, epigenomics, and metabolomics, opening new avenues in spatial biology.

Several excellent reviews have previously addressed components of this landscape. Moses and Pachter [30] cataloged ST technologies through an evolutionary lens; Zeng et al. [31] and Heydari and Sindi [32] focused on statistical and DL methods for ST; Luo et al. [33] reviewed deep learning for multimodal ST integration; and Coleman et al. [34] discussed AI’s broader role in spatial omics. In comparison with previous reviews (Table 1), this work covers AI-related methods across the complete ST workflow, including preprocessing, downstream analysis, multimodal integration, and interpretation. It also evaluates major ST databases and repositories, summarizes available benchmarking evidence, and examines the current evidence and limitations associated with foundation models and large language models (LLMs).

**Table 1:** Comparison of recent reviews of spatial transcriptomics technologies and computational analysis.

Review	Primary scope	AI/ML/DL	Benchmark synthesis	Databases	Multi-omics	Foundation models/LLMs	Distinction from present review
Moses and Pachter	Evolution of ST technologies	Limited	No	Limited	Partial	No	Primarily technology-focused
Zeng et al.	Statistical and ML methods	Yes	Partial	No	Limited	No	Focuses on established analytical methods
Heydari and Sindi	Deep learning in ST	DL-focused	Partial	No	Partial	No	Focuses specifically on DL
Luo et al.	DL for multimodal integration	DL-focused	Partial	No	Yes	Limited	Concentrates on multimodal integration
Coleman et al.	AI in spatial omics	Yes	Limited	No	Yes	Partial	Broad perspective rather than full workflow
Present review	AI across the complete ST workflow	Yes	Yes	Yes	Yes	Yes	Integrates platform context, databases, benchmarks, AI-method selection, and LLM readiness

Thus, this review focuses on the intersection of computational methods and STs, summarizing various types of ST technologies and the complex, large-scale datasets generated by these methods. After discussing the integration of ML, DL, and AI-based computational tools for analyzing ST data, the review also covers multi-omics integration pipelines. We have highlighted the applications of ST in various research domains and concluded with a discussion of the challenges, limitations, and future directions that will shape the development of spatial omics in the years to come. This review provides a thorough overview of how ST technologies advance biomedical research and expand our understanding of both health and disease.

## Spatial transcriptomics technologies

STs comprises experimental methods that measure RNA expression while retaining spatial coordinates within a tissue section. Depending on the platform, measurements may represent individual transcripts, segmented cells, spatial bins, beads, or multicellular capture spots. ST technologies can primarily be divided into two categories: imaging-based and sequencing-based approaches [16, 35]. Imaging-based ST methods provide direct visualization of RNA molecules through advanced microscopy techniques, whereas sequencing-based ST methods harness spatially barcoded arrays, enabling high-throughput RNA profiling that offers comprehensive insights into gene expression [36]. Recent advancements in integrating ST with other omics data, such as proteomics, epigenomics, and metabolomics, provide deeper insights into tissue biology [30, 37]. By maintaining the native tissue context, these integrated multimodal approaches enhance our understanding of cellular organization, the intricate dynamics of the TME, and the underlying mechanisms of various diseases [38].

### Imaging-based ST methods

Imaging-based ST methods utilize advanced approaches, including fluorescence microscopy and molecular probing, to directly visualize RNA molecules within intact tissues without the need for RNA extraction [36]. The RNA is fixed within the tissue sections and then labeled with special fluorescent tags or barcoded oligonucleotides that precisely bind to targeted RNA molecules. They are subsequently used for detailed imaging of the transcripts through repeated cycles of hybridization and sequencing, resulting in the quantification of RNA transcripts [16, 39].

The imaging-based methods are typically divided into two: *in situ* hybridization (ISH) and *in situ* sequencing (ISS). ISH methods utilize nucleic acid hybridization to visualize gene expression directly within fixed tissues or cells. It is done by employing fluorescently labeled DNA or RNA oligonucleotides, which are specifically designed to bind to their complementary target RNA molecules [40, 41]. To determine the spatial distribution of these transcripts, fluorescence microscopy is used to detect the signals of the attached probes, ensuring the precise mapping and quantification of gene expression within its native cellular environment. On the other hand, ISS is a highly specific and sensitive technique that offers multiple levels of molecular specificity and multiplexing, allowing for the analysis of several hundred transcripts per sample [42]. It allows the localization and quantification of over 100 transcripts simultaneously at subcellular resolution, all within a single tissue section using padlock probes, rolling-circle amplification, and barcode decoding [43].

#### *In situ* hybridization

Various technologies fall under ISH, such as single-molecule Fluorescence *in situ* Hybridization (smFISH), which utilizes multiple short probes targeting a single transcript, producing fluorescence corresponding to the individual RNA molecules [44]. It provides precise detection of targeted RNA molecules with high sensitivity and subcellular spatial resolution, however it can only target a few genes at a time and has limited multiplexing [45]. Cyclic smFISH (osmFISH) is an advanced version of the smFISH technique that performs iterative rounds of probe hybridization, imaging, and removal to quantify individual RNA molecules in tissue sections [46]. This non-barcoded approach can handle multiplexing of hundreds of genes with higher accuracy and sensitivity [47]. However, the repeated cycles required for multiplexing make it a time-consuming and labor-intensive method. Sequential FISH (seqFISH) is another multiplexing technique that utilizes multiple rounds of sequential hybridization and imaging of barcoded probes to detect multiple genes in a single cell, while preserving spatial information [48]. seqFISH+ is an even more powerful method that can target 10 000 genes at a time, achieving almost transcriptome-wide coverage while improving sensitivity and spatial resolution [49]. Multiplexed Error-Robust FISH (MERFISH) enables highly multiplexed and error-robust detection of RNA transcripts in individual cells. It assigns unique, error-robust binary barcodes to each RNA molecule. The barcodes are then read in sequential rounds of hybridization and imaging, allowing thousands of transcripts to be imaged simultaneously [50, 51].

There has been a rapid advancement in the commercialization of ISH-based ST tools, providing highly multiplexed RNA detection with enhanced spatial resolution. For instance, NanoString’s CosMx Spatial Molecular Imager (SMI) allows high-plex cyclic smFISH-based detection of up to 1000 RNA targets and over 100 proteins at single-cell resolution [52]. In contrast, GeoMx Digital Spatial Profiler (DSP) offers region-of-interest-based profiling of both RNAs and proteins [53]. Other key platforms include Vizgen’s MERSCOPE; Akoya Bioscience’s PhenoCycler-Fusion [54], 10× Genomics’ Xenium with integrated ISS [55]; RNAscope from Advanced Cell Diagnostics (ACD) Biotechne [56]; and Resolve Biosciences’ Molecular Cartography [57]. These platforms facilitate advanced studies in spatial biology at single-cell and subcellular resolutions, enhancing our understanding of tissue architecture and cellular interactions.

#### *In situ* sequencing

ISS methods reverse transcribe the RNA molecule into complementary DNA (cDNA) directly within the tissues, followed by *in situ* amplification and sequencing [58]. This approach enables the identification of transcripts based on their nucleotide sequence rather than relying on predefined probes. Several ISS-based methods have been developed so far. Padlock probe-based ISS is a targeted sequencing method that utilizes padlock probes that hybridize to cDNA [59]. These probes are then enzymatically ligated to form a circularized DNA molecule, which is amplified through rolling circle amplification (RCA) to form DNA nanoballs. Lastly, the amplified products are interpreted through repeated cycles of sequencing-by-ligation, thereby identifying transcripts directly within the tissues. This approach enables the multiplexed detection of hundreds of genes, as well as the identification of single-nucleotide variants, with high specificity and sensitivity [60].

Another method, FISSEQ [61, 62], enables unbiased transcriptome-wide profiling without requiring a predefined set of probes. It amplifies cDNA using the RCA method, followed by sequencing-by-ligation chemistry with repetitive cycles of hybridization, imaging, and signal removal to accurately read RNA transcripts. However, its relatively low sensitivity and technical complexity have led to the development of more improved ISS-based methods. STARmap employs a padlock probe-based approach to amplify RNA molecules without reverse transcription. It combines hydrogel-tissue chemistry with Specific Amplification of Nucleic Acids via Intramolecular Ligation (SNAIL) probes and Sequencing with Error-reduction by Dynamic Annealing and Ligation (SEDAL). The method provides high-resolution 3D ST profiling within thick tissue samples and has a high multiplexing capacity to profile hundreds to thousands of genes at single-cell resolution simultaneously [63]. ExSeq integrates ISS with expansion microscopy to physically enlarge tissues. This approach enhances spatial resolution and reduces optical crowding, allowing for the precise mapping of RNA transcripts within subcellular localizations in intact tissues [64]. BaristaSeq is a highly efficient method developed to improve the accuracy of traditional ISS. This is achieved by optimizing the design of padlock probes in conjunction with Illumina sequencing-by-synthesis chemistry. BaristaSeq demonstrates a five-fold increase in both signal strength and sequencing accuracy, making it suitable for targeted ST applications like cell lineage tracing, neural circuit mapping, and cancer tissue profiling, all with high spatial precision [65].

Overall, the ISH methods provide higher sensitivity and resolution for identifying targeted transcripts, while ISS methods offer detailed sequence-level information and greater scalability. Together, these approaches enhance gene expression analysis within tissues by combining high-resolution spatial mapping with broader transcriptome coverage.

### Sequencing-based ST methods

Sequencing-based methods in ST employ NGS along with spatially barcoded arrays on substrates such as slides or chips, to capture RNA molecules directly from intact tissue samples. In contrast to imaging-based methods, sequencing-based approaches offer unbiased profiling and high scalability across large tissues and diverse sample types [10]. Some notable research and commercial-based platforms include 10× Genomics Visium [66], Slide-seq/Slide-seqV2 [50, 51], HDST [67], DBiT-seq [68], Stereo-seq [69], Seq-Scope [70], and PIXEL-seq [71].

10× Genomics Visium is a widely used commercial platform for transcriptome-wide coverage directly from intact tissue samples. It utilizes specialized glass slides with thousands of capture spots each ~55 μm in diameter [72]. Every spot contains a unique spatial barcode and poly (dT) tails for capturing RNA transcripts. It is compatible with both formalin-fixed paraffin-embedded (FFPE) and fresh-frozen tissue samples. The samples are placed on the barcoded glass slides and sequenced using NGS, resulting in a comprehensive spatial map of gene expression across the whole transcriptome, which has been extensively applied in cancer biology, neuroscience, and developmental biology research [73].

Slide-seq/Slide-seqV2 involves the hybridization of tissue samples onto DNA-barcoded beads, which are ~10 μm in diameter. These beads have a fixed spatial location. Each hybridized bead is sequenced to determine the spatial resolution of RNA transcripts, while the HDST method advances spatial resolution to the sub-cellular level (~2 μm). DBiT-seq is a multi-omics platform that facilitates the co-detection of RNA transcripts and proteins using microfluidic barcoding. Stereo-seq, developed by BGI Genomics, leverages a patterned array of DNA nanoballs (DNB) with nanometer-scale barcoding, enabling subcellular resolution of ~500 nm [69]. Seq-scope offers ultra-high resolution (~0.5–0.8 μm) with nanoscale detail, allowing for spatial transcriptome profiling and sub-cellular level visualization of gene expression. Finally, Pixel-seq employs patterned arrays of spatially barcoded primers to achieve high sensitivity compared to Slide-seq and HDST. It allows uniform spatial sampling, which reduces bias and offers high spatial resolution for mapping fine-scale tissue structures.

Sequencing-based ST methods offer higher scalability and unbiased, high-throughput whole-transcriptome profiling, allowing reconstruction of tissue-wide molecular atlases from intact tissue samples. However, a significant limitation is their low spatial resolution compared to imaging-based methods, making it challenging to achieve single-cell resolution.

### Multi-modal, hybrid, and emerging ST platforms

Beyond the established imaging- and sequencing-based categories, a new generation of hybrid platforms is emerging by combining *in situ* sequencing with NGS barcoding, or by co-profiling RNA with proteins, chromatin accessibility, and metabolites at spatial resolution. For example, DBiT-seq [68] and its successor xDBiT [74], Matrix-seq [75], and MAGIC-seq [76] co-detect RNA and proteins using microfluidic barcoding on the same tissue section. Spatial epigenomics platforms -including ATAC-seq and spatial CUT&Tag- are now being combined with ST to co-profile chromatin accessibility and gene expression from the same tissue. Spatial metabolomics approaches using mass spectrometry imaging and desorption electrospray ionization are increasingly being registered with ST datasets to link metabolic states to transcriptional programs [77].

While PIXEL-seq achieves single-molecule RNA detection alongside spatial barcoding [77], recent platforms such as 10× Genomics Visium HD, released in 2024, extend barcoded array resolution to 2 μm, approaching single-cell resolution for sequencing-based methods. Conversely, Xenium integrates ISS with computational spot-to-cell assignment for direct single-cell localization.

Other advancements include microdissection-based technologies, such as laser-capturing microdissection [78, 79], Tomo-seq [80], and Geo-seq [81], which provide specific positional information that helps reconstruct 3D tissue structures. Approaches such as ProximID [82], PIC-seq [83], and ClumpSeq [84] offer high-resolution molecular analysis by partially dissociating tissue samples while maintaining their spatial context. Optical barcoding methods, NICHE-seq [85], ZipSeq, and TIVA [86], use photoactivated oligonucleotide barcodes or TIVA tags to label and profile regions of interest within the native tissue environment.

These hybrid approaches greatly expand the biological information obtainable from a single tissue section but introduce additional computational challenges related to data integration, resolution mismatches, and multi-modal batch correction. A technical comparison of representative ST platforms is provided in Table 2, where throughput and sensitivity categories are qualitative because direct comparisons are affected by tissue type, sample preparation, panel design, sequencing depth, image processing and segmentation, and platform specifics.

**Table 2:** Technical comparison of representative ST platform categories.

Platform category	Representative examples	Resolution	Coverage	Throughput	Relative sensitivity	Principal advantage	Principal limitation	Suitable use
Imaging-based targeted RNA	MERFISH, Xenium, CosMx	Single-cell to subcellular	Targeted panels	Moderate to high	Generally high for targeted genes	Precise cellular localization	Requires predefined panels	Cell-state mapping and tissue niches
Array-based sequencing	Visium, Visium HD	Spot-level to near-cellular	Whole transcriptome	High tissue-area coverage	Moderate and tissue-dependent	Unbiased discovery	Diffusion, sparse counts, or mixed-cell measurements	Tissue-wide discovery
Bead- or array-based high resolution	Slide-seqV2, Stereo-seq, Seq-Scope	Near-cellular to subcellular	Whole transcriptome	High, with substantial computational demands	Platform-dependent	High spatial granularity	Sparse data and intensive computation	Fine-scale tissue architecture
Multimodal spatial assays	DBiT-seq, Spatial-CITE-seq	Platform-dependent	RNA plus proteins or chromatin	Low to moderate	Modality-dependent	Joint molecular characterization	Resolution mismatch and complex integration	Spatial multi-omics

## Spatial transcriptomic data resources

The advent of ST technologies has led to the generation of a vast amount of heterogeneous data across tissues, species, and disease contexts. However, the produced datasets were dispersed across the literature and are not systematically organized. To overcome this limitation, various specialized databases and repositories have been developed to make these datasets easily accessible. These resources provide an infrastructure that combines raw, original data with advanced visualization tools to facilitate data sharing, standardization, and large-scale analyses.

Major databases and repositories include DeepSpaceDB, a newer platform that hosts thousands of samples from human and mouse tissues, enabling advanced interactive analysis and promoting large-scale cross-study integration [87]. SOAR is a comprehensive database hosting largest number of ST samples, i.e. 4767 samples with spatial coordinates from 13 species across 58 tissue types [88]. SORC is a cancer-specific ST repository that contains 82 ST datasets across 17 cancer types, comprising over 722 899 spots for 269 tissue slices. It is an open-source, user-friendly platform that facilitates the integration of multi-omics data [89]. STOmicsDB is another comprehensive database for sharing, analyzing, and visualizing ST data. It contains more than 300 manually curated datasets representing 17 species [90]. CROST stores 544 high-quality ST datasets and 48 043 tumor-related spatially variable genes (SVGs) spanning various species, organs, and diseases [91]. SPASCER contains datasets from 43 studies, including 1082 sub-datasets from 16 organ types across four species [92]. Aquila is a spatial omics database and analysis platform, comprising 110 datasets derived from 30 diseases, incorporating more than 6500 regions of interest, and 15.7 million cells. The platform integrates studies across ST and proteomic analyses, as well as 2D and 3D experimental frameworks, and distinct technologies [93]. SODB is a repository that allows cross-platform comparisons and benchmarking. It maintains data from around 3000 experiments from more than 25 different spatial omics technologies [94]. SpatialDB is one of the earliest databases for spatially resolved transcriptomes. It encompasses 24 datasets and 305 sub-datasets across five species generated by eight different ST methodologies [95].

All these repositories emphasize standardized metadata, submission frameworks, and data formats to facilitate FAIR (Findable, Accessible, Interoperable, Reusable) data sharing and reusability in ST [96, 97]. These databases are publicly available and feature user-friendly web interfaces, enabling non-computational experts from various biological fields to easily access and analyze the information. List of databases and repositories curated for comprehensive ST data collection are listed in Table 3.

**Table 3:** List of databases and repositories curated for comprehensive ST data collection.

Database (Link)	Year	Samples or datasets	Species	Disease focus	Best use case	Limitations	Ref.
DeepSpaceDB (https://genomics.virus.kyoto-u.ac.jp/deepspacedb/)	2025	2700+ samples	Human and mouse	Broad	Interactive in-depth analysis, large-scale integration	Visium and Xenium data deposition only	[87]
SOAR (https://soar.fsm.northwestern.edu/)	2025	4767 samples	13 species and 58 tissue types	Cross-disease, drug discovery	Large-scale cross-species analysis; integrates protein interaction networks	Limited to available public datasets	[88]
SORC (http://bio-bigdata.hrbmu.edu.cn/SORC/)	2024	82 datasets (17 cancer types)	Human	Cancer	Oncology-focused multi-omics analysis	Only cancer-specific	[89]
STOmicsDB (https://db.cngb.org/stomics/)	2024	378 datasets	17 species	Broad	Data sharing and interactive visualization across species	Limited downstream analysis tools	[90]
CROST (https://ngdc.cncb.ac.cn/crost)	2024	544 datasets	Multiple	Cancer	Tumor-related SVG discovery	Focused on tumor tissues	[91]
SPASCER (https://ccsm.uth.edu/SPASCER/)	2023	43 studies; 1082 sub-datasets	4 species	Broad	Single-cell resolution annotation	Smaller sample size than SOAR	[92]
Aquila (https://aquila.cheunglab.org/)	2023	110 datasets; 6500+ ROIs	Multiple	30 diseases	2D/3D spatial + proteomic integration	Requires registration for download	[93]
SODB (https://gene.ai.tencent.com/SpatialOmics/)	2023	3000+ experiments	12 species	Broad	Cross-platform benchmarking	Heterogeneous data formats	[94]
Spatial DB (http://spatialomics.org/SpatialDB/)	2020	24 datasets	5 species	Broad	Earliest resource; good for legacy comparisons	Small, infrequently updated	[95]

### Database selection guide

As listed in Table 3, for cancer research, SORC provides the most curated cancer focused ST data with multi-omics integration capability while CROST is ideal for SVG-centered analyses. For neuroscience, STOmicsDB and SOAR contain large brain atlas datasets. For cross-species and plant biology, SOAR and STOmicsDB support the widest species range. For benchmarking computational tools, SODB covers the most diverse platform types. When integrating data from multiple databases, users should ensure spatial coordinate systems are harmonized and apply batch correction tools. Note that data format heterogeneity (HDF5, Parquet, CSV) may require custom conversion scripts before any analysis.

### Challenges of ST data resources

While providing vastly rich datasets, the STs repositories contain datasets generated using heterogeneous platforms, spatial resolutions, transcriptome coverages, file formats, coordinate systems, segmentation procedures, and metadata conventions (Fig. 1). These differences impair interoperability and introduce batch effects, annotation drift, feature mismatch, and possible train-test contamination in AI studies. The consequences include unreliable benchmarking, limited reproducibility, weak cross-platform transfer, and substantial computational burden. Common data models, controlled vocabularies, provenance tracking, dataset versioning, study-level train-test separation, platform-aware harmonization, and FAIR metadata are therefore needed to support robust integration and AI-model validation.

**Figure 1:**
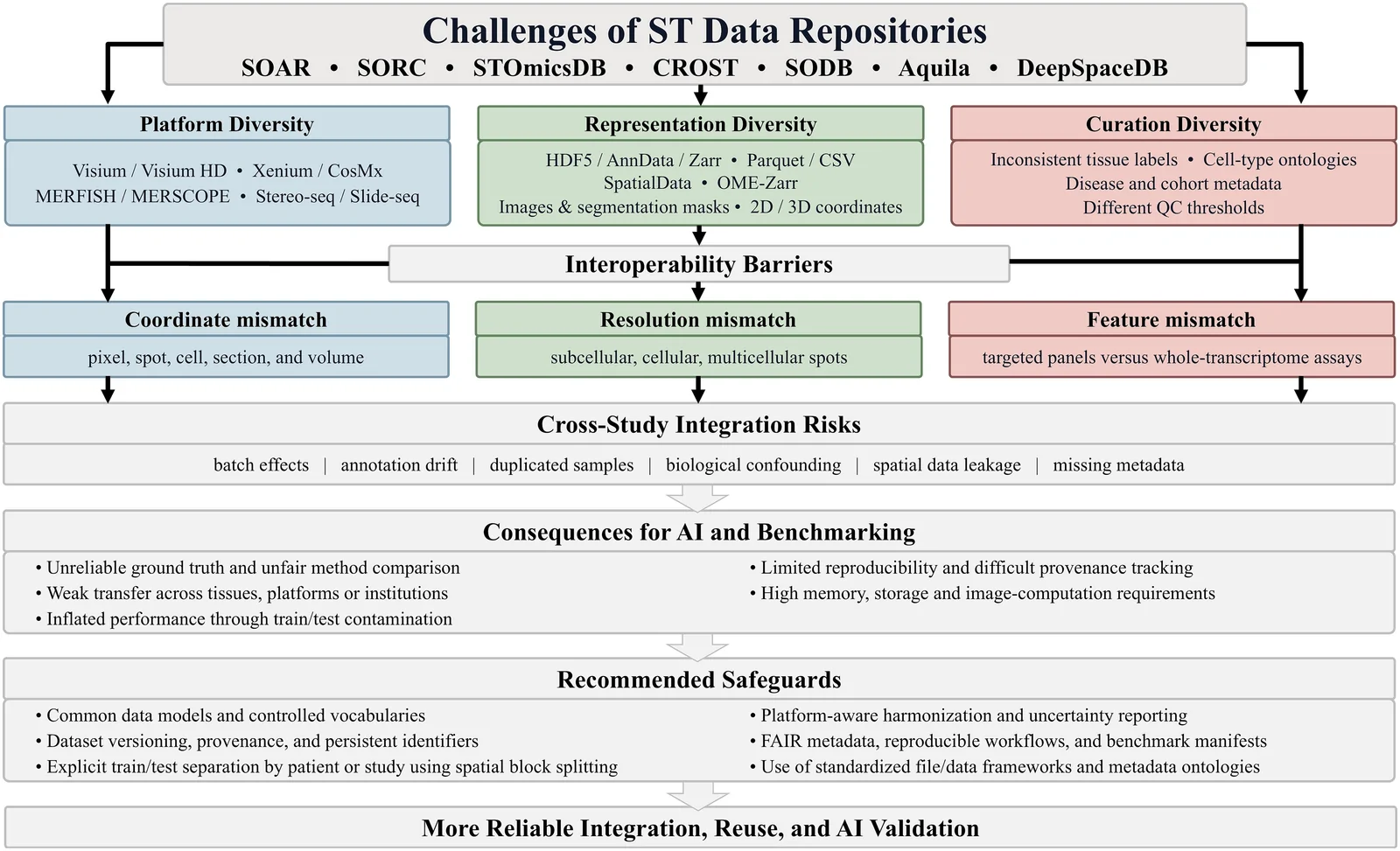
Challenges of ST data resources. Spatial transcriptomics repositories contain datasets generated using heterogeneous platforms, spatial resolutions, transcriptome coverages, file formats, coordinate systems, segmentation procedures, and metadata conventions. These differences impair interoperability and introduce batch effects, annotation drift, feature mismatch, and possible train-test contamination in AI studies. The consequences include unreliable benchmarking, limited reproducibility, weak cross-platform transfer, and substantial computational burden. Common data models, controlled vocabularies, provenance tracking, dataset versioning, study-level train-test separation, platform-aware harmonization, and FAIR metadata are therefore needed to support robust integration and AI-model validation.

## Spatial transcriptomic data analysis

The primary objective of ST analysis is to combine gene expression data with spatial location information to extract topological insights from the biological specimen. The data analysis process can be broadly divided into pre-processing and downstream analysis [30, 98]. The two stages are tightly coupled, where data quality, normalization strategy, and coordinate representation established during pre-processing directly determine the reliability of subsequent downstream analysis in clustering, deconvolution, and interaction analyses (Fig. 1 and Table 4). Various software packages have been developed to streamline the workflow, with numerous reviews highlighting the methods and their functionalities [31, 32, 99–102]. Table 4 lists the computational tool examples used at each analysis step depicted in Fig. 2 and summarizes the main approaches and strengths of these methods. It also lists some limitations of the current tools. To maintain readability of the current work, the main text discusses only representative, benchmarked, or methodologically distinct approaches while comprehensive tool inventories and the software links are provided in Supplementary Tables 1–11.

**Table 4:** Representative computational methods for ST analysis.

Analysis stage	Approach	Representative tools	Typical input	Key strength	Limitation
Image preprocessing	DL (CNN/U-Net)	DeepBlink, Cellpose, GHIST, CellProfiler	Raw fluorescence images	High-throughput cell segmentation	Platform-specific tuning; possible GPU requirement
Batch correction	Embedding/GNN	Harmony, Crescendo, SpaCross, SPIRAL	Expression + spatial coordinates	Multi-sample integration	Over-correction of biological variation
Dimensionality reduction	Graph/Bayesian	STAGATE, SpatialPCA, STAMP, GraphPCA	Gene expression matrix	Spatially aware embeddings	Parameter sensitivity
Spatial clustering	GNN/Bayesian	GraphST, BayesSpace, MAEST, SC-MEB	Low-dimensional embeddings	Spatially coherent domain detection	Varying tissue type benchmarks
Cell-type deconvolution	Probabilistic/DL	Cell2location, RCTD, CARD, DestVI	ST spots + scRNA-seq reference	Cell-type proportion estimation	Reference quality dependence
SVG detection	Statistical/DL	HEARTSVG, Castl, GAADE, DESpace	Spatial expression matrix	Gene pattern discovery	Scalability at whole-transcriptome level
Cell–cell communication	Ligand-receptor/GNN	CellChat, NicheNet, SpatialDM, CytoSignal	Expression + spatial coordinates	Intercellular interaction inference	Ligand-receptor database incompleteness
Spatial and temporal analysis	Graph learning	spVelo, Cell2fate, SpaTrack, SpaTalk	ST / scRNA-seq	RNA velocity and trajectory inference	Sensitivity to prior processing and noisy data
Multi-omics integration	GNN/DL	NicheCompass, BANKSY, Tangram, MOFA+	Multi-modal spatial data	Cross-modality biological insight	Data heterogeneity; missing values
3D reconstruction	DL	So3D, X-Pression, isoST, SpaBatch	ST + serial 2D or 3D coordinates	3D tissue architecture reconstruction	Computational cost; scalability; lack of ground truth
Visualization	Interactive/web	Vitessce, Giotto, Squidpy, SpatialView	Any ST output	Interpretable exploration	Scalability for large datasets

**Figure 2:**
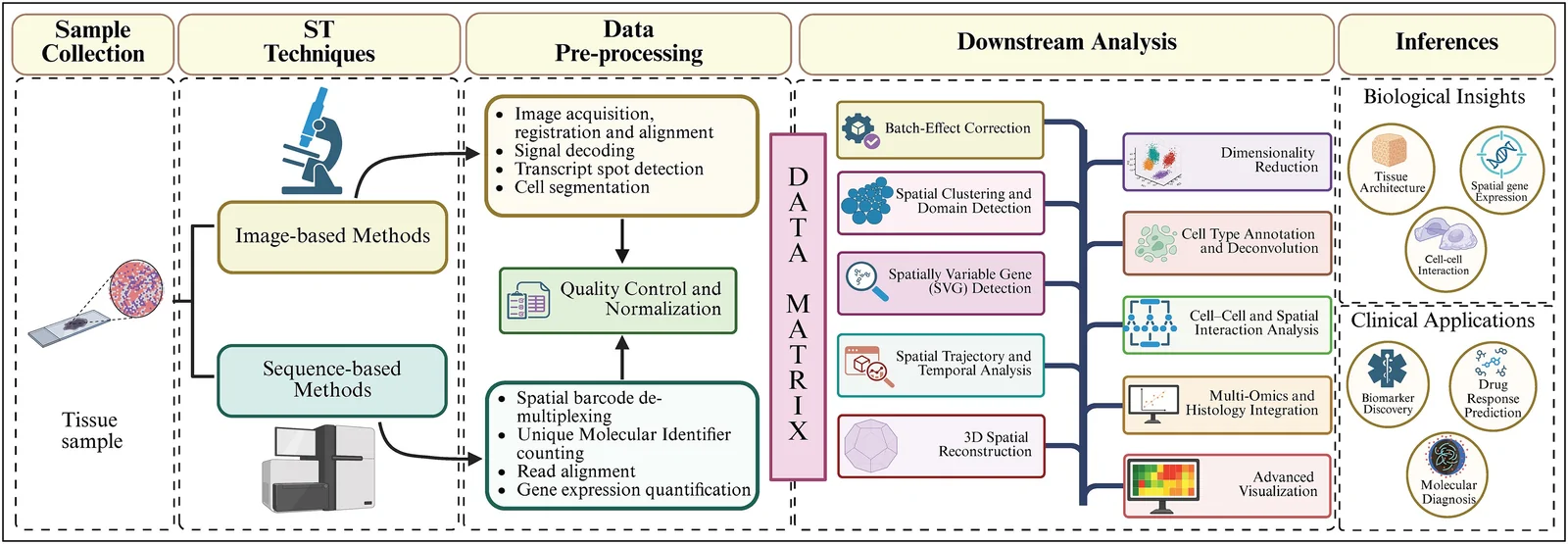
Overview of spatial transcriptomics data analysis workflow. The workflow begins with tissue sample acquisition, followed by two parallel pre-processing streams: imaging-based pre-processing (applicable to ISH/ISS platforms such as MERFISH, Xenium, and CosMx), which encompasses image acquisition, registration, transcript spot identification, cell segmentation, and quality control; and sequence-based pre-processing (applicable to NGS-barcoded platforms such as Visium, Slide-seq, and Stereo-seq), which encompasses spatial barcode demultiplexing, read alignment, and gene expression matrix generation. Both streams converge on a normalized, spatially resolved gene expression matrix (the Data Matrix). This matrix serves as the input for the 10 steps of downstream analysis. Each downstream step builds on the previous such that dimensionality reduction compresses data to preserve structure for clustering; clustering defines spatial domains that contextualize SVG detection; SVG and interaction analyses reveal functional organization; and trajectory inference links spatial patterns to dynamic biological processes. The integrated outputs feed into application domains including cancer biology, neuroscience, immunology, infectious disease, and drug discovery/precision medicine, ultimately aiming for clinical translation through spatially informed biomarker development and patient stratification. The figure is created in BioRender. Gundamaraju, R. (2026) https://BioRender.com/vevxu78 (licensed under CC BY 4.0).

### AI, ML, and DL: a definitional framework in the context of ST

AI is the broadest term, referring to any computational system designed to perform tasks that would otherwise require human-like reasoning or pattern recognition. ML is a subset of AI in which models learn from data without being explicitly programmed. It encompasses methods such as linear regression, support vector machines, and random forests. DL is a subset of ML that uses multi-layer neural networks to learn hierarchical representations from large datasets. It includes convolutional neural networks (CNNs), graph neural networks (GNNs), variational autoencoders (VAEs), and transformer architectures. LLMs are another subcategory of DL, specifically transformer-based models trained on large-scale text collections. In ST, these computational tiers map to the analytical pipelines: classical statistical and machine learning methods (including PCA, clustering, and regression) dominate preprocessing; DL (GNNs, CNNs, VAEs) drives domain identification, deconvolution, and trajectory inference; and LLMs are emerging for interactive, interpretive, and integrative tasks.

### Data pre-processing: image-based pre-processing and sequence-based pre-processing

In ST data analysis, pre-processing is essential to ensure data quality and reliability before any in-depth analysis. The first step is quality control to remove low-quality data, followed by normalization to ensure accurate biological findings during downstream analysis. Depending on which type of ST method is used, data pre-processing is classified into two categories: imaging-based pre-processing for ISH and ISS-based methods, which capture RNA molecules using fluorescent imaging, and sequencing-based pre-processing for NGS-based spatial barcode methods.

Imaging-based pre-processing methods in ST are a critical set of computational and image analysis workflows designed to convert raw image data into reliable spatial gene expression information. These methods are primarily applied to imaging-based ST technologies, such as ISH, ISS, and fluorescence-based techniques. The workflow includes image acquisition, image registration and alignment, transcript spot identification, signal decoding, cell segmentation, quality control, and normalization [10, 103]. For image acquisition, raw images from different hybridization methods are corrected for illumination artifacts, focus drift, autofluorescence, and background noise to ensure accurate signal detection. Tools like ImageJ/Fiji, Napari, Elastix, and BigStitcher are commonly used for basic image adjustments, such as contrast correction, noise reduction, and stitching [104–107].

Followed with image registration and alignment, the microscopy images are precisely aligned to create a unified spatial coordinate system [108]. After alignment, transcript spot identification and localization are performed to detect individual RNA molecules in tissue images, using tools like DeepBlink, a representative deep-learning method [109]. Cellpose and StarDist are widely used segmentation baselines in the next step [110, 111]. Quality control and normalization are integrated throughout the imaging pipeline, applying spatially aware smoothing and noise reduction techniques [112]. Recent advances incorporate DL and AI methods to enhance pre-processing accuracy by integrating histology features with spatial gene expression patterns. For instance, GHIST is a DL-based method trained on millions of histological images to extract morphological features, improving spatial domain identification [113].

Sequence-based pre-processing applies to NGS-based technologies that generate sequencing data with spatial barcode information, including Visium, Slide-seq, and Stereo-seq. The goal is to transform raw NGS reads into spatially resolved gene expression matrices through various computational pipelines. The major steps for sequencing-based pre-processing include spatial barcode mapping and demultiplexing, read alignment and gene quantification, histological image processing, and relating gene expression to tissue images [103, 114]. The workflow begins with spatial barcode processing and demultiplexing, utilizing standard pipelines such as Space Ranger for comprehensive barcode mapping, read alignment, and expression quantification [115]. STtools aligns the reads to a reference genome and maps them back to their corresponding spatial coordinates [116]. For read alignment and transcript quantification, tools like STAR and HISAT2 provide high-accuracy gapped alignment of reads [117, 118]. For histological image integration, packages are used to spot coordinates onto H&E-stained images, facilitating spot-to-tissue mapping and extracting morphological features for downstream analysis.

Overall, imaging-based and sequence-based pre-processing ensures the generation of high-quality, spatially resolved transcriptomic datasets, providing a strong foundation for downstream spatial analyses. So, selecting tools according to the specific error source, such as segmentation or transcript assignment, is advantageous rather than applying a single general image-analysis package to the complete workflow. Although Cellpose or StarDist can provide useful baselines, assay-aware methods may be preferable when transcript coordinates, membrane markers, or tissue-specific training data are available. In sequence-based pre-processing, using platform-specific pipelines for initial barcode and coordinate processing makes sense, usually with parameter tuning. Importantly, inspecting the spatial distribution of quality-control metrics can prevent removal of biologically meaningful low-RNA regions. The tools of data pre-processing for ST are enlisted in the Supplementary Table 1.

### Downstream analysis

After data pre-processing, where raw images and sequencing reads are transformed into a gene expression or **Data Matrix**, the next step is downstream analysis (Fig. 1). The process employs various computational and statistical methods to extract biological or clinical insights from the processed ST data. The key steps include batch-effect correction, dimensionality reduction, spatial clustering and domain detection, cell-type annotation and deconvolution, detection of SVGs, spatial interaction analysis, spatial trajectory and temporal analysis, integration with omics and histology data, 3D spatial reconstruction, and advanced visualization [119, 120]. A wide range of advanced ML, DL, and AI tools has been developed for these applications, which are used to interpret ST data and understand the spatial organization of genes, cells, and tissues that underlie complex biological systems and disease processes.

The selection of computational methods should be guided by the biological question, data-generating platform, spatial resolution, availability of histological images and single-cell references, modality matching, and computational scale. Figure 3 provides an evidence-guided decision framework connecting these dataset characteristics to the principal analytical tasks and representative method classes. Rather than identifying a universally optimal tool, the framework emphasizes task-appropriate selection, comparison of alternative approaches, sensitivity assessment, uncertainty reporting, and independent biological validation.

**Figure 3:**
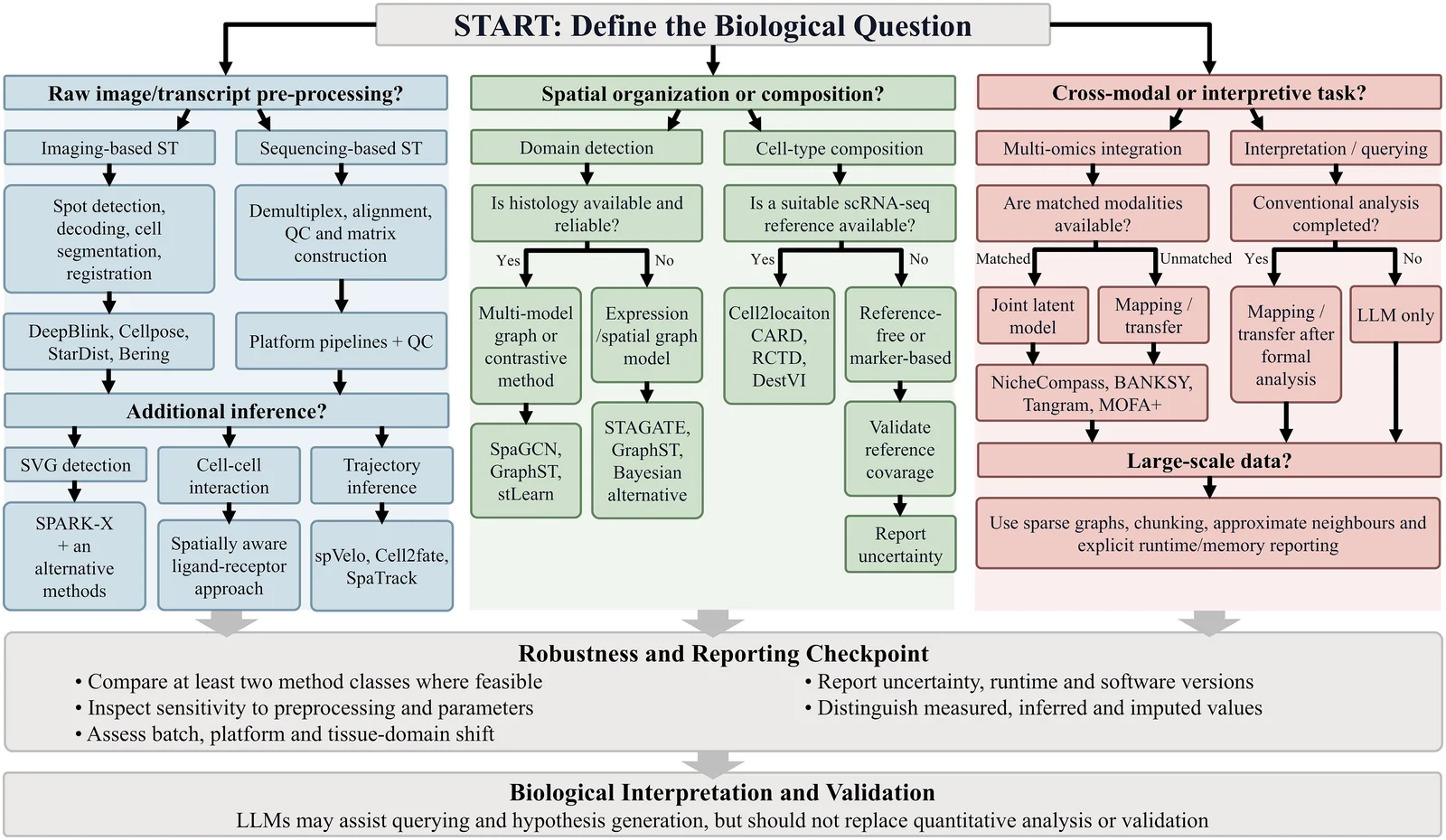
Evidence-guided selection of AI and computational methods for spatial transcriptomics. Method selection should begin with the biological question, data-generating platform, available modalities, spatial resolution, and reference data, rather than with the popularity of a particular model or tool. Imaging-based workflows emphasize transcript detection, decoding, registration, and cell segmentation, whereas sequencing-based workflows require barcode processing, alignment, quality control, and generation of spatial expression matrices. Spatial-domain and deconvolution methods should be selected according to histology availability, reference quality, tissue complexity, and desired uncertainty estimates. Cross-modal integration requires explicit consideration of matched versus unmatched samples and resolution mismatch. LLMs are positioned after quantitative analysis as assistive tools for querying, annotation, and hypothesis generation. Regardless of the selected method, the workflow should include sensitivity analysis, reporting of preprocessing and parameter settings, and validation of major findings using independent data or orthogonal evidence where feasible.

#### Batch-effect correction

Batch effects are unwanted variations caused by differences and inconsistencies in experimental conditions, such as sample preparation (using different slides or tissue sections) or sequencing platforms and protocols [119, 121]. Such effects can hide the actual biological signals and affect the downstream analysis. Therefore, batch-effect correction is an essential first step for eliminating technical variations that can mislead results and impact research outcomes. Conventional approaches such as Harmony provide a non-spatial integration baseline, whereas spatialMNN and graph-based frameworks such as SPIRAL or SpaCross incorporate spatial neighborhood information [122–125]. Some of these methods were initially developed for scRNA-seq but are now widely used where they can effectively process ST data by utilizing spatial coordinates, expression profiles, and neighborhood graphs. The goal is to minimize batch effects while preserving biological architecture, enabling accurate integration of multiple samples. At this stage, users should identify the expected technical and biological sources of variation before applying the correction and compare corrected and uncorrected analyses to verify that tissue architecture, rare populations, condition effects, and spatial gradients remain detectable. Various tools developed for batch effect correction are listed in Supplementary Table 2.

#### Dimensionality reduction

Dimensionality reduction is the process of transforming high-dimensional data into lower-dimensional data that preserves key information while reducing the number of features. In ST, it condenses the gene expression profiles into low-dimensional embeddings that also retain the spatial context. PCA remains a transparent and computationally efficient baseline, but it does not explicitly model spatial relationships. Spatially aware methods incorporate neighborhood graphs or spatial covariance: SpatialPCA uses a probabilistic framework, STAGATE learns graph-attention embeddings, and STAMP uses topic modeling to generate spatially informed and interpretable latent factors [126–128]. Also, tools like SpaGCN [129] combine dimension reduction with spatial neighborhood analysis, visualization, and clustering, thereby offering integrated workflows for comprehensive exploration of ST data. Regardless of the tool used, the appropriate representation depends on the downstream objective, and embeddings should be evaluated by stability, biological marker coherence, spatial continuity, and downstream reproducibility rather than visual separation alone. The details of many dimensionality reduction tools are given in Supplementary Table 3.

#### Spatial clustering and domain detection

Spatial clustering, also referred to as spatial domain identification, facilitates the identification of distinct tissue regions and microenvironments that share similar gene expression patterns and spatial organization. These spatial domains are the regions within tissues where cells exhibit similar molecular signatures, cellular interactions, and most likely perform related biological functions. The domains are computationally identified by spatial clustering, where coherent regions are grouped based on their transcriptional profile similarity and spatial organization [130, 131].

Existing approaches include graph-based representation learning, Bayesian spatial modelling, and conventional clustering. Conventional community detection algorithms, like Louvain and Leiden [132], utilize k-nearest neighbor graphs on low-dimensional embeddings and partition them into spatially coherent clusters. STAGATE and GraphST learn spatially informed graph embeddings, SpaGCN can incorporate histological information, and BayesSpace uses probabilistic spatial regularization [128, 129, 133, 134]. Multi-sample frameworks extend domain identification to aligned or jointly modeled tissue sections. Recent advancements to SpaGCN and stLearn [135] integrate morphological features derived from histological images (such as H&E staining) and transcriptomic data, employing graph convolution and smoothing algorithms to enhance spatial domain identification [135]. The details of these tools are given in Supplementary Table 4.

These existing methods differ substantially in how they weight spatial versus transcriptomic signals. Graph-based methods such as GraphST and STAGATE have performed competitively in several benchmarks, but their results depend on graph construction, preprocessing, platform, and tissue architecture. BayesSpace provides probabilistic spatial regularization, whereas Louvain or Leiden clustering offers computationally efficient non-spatial baselines. Selection should be based on the analytical objective, dataset size, spatial resolution, histology availability, and required form of uncertainty assessment.

#### Cell-type annotation and deconvolution

Cell-type annotation and deconvolution are essential steps in ST data analytics, tackling the challenge of identifying distinct cell types and evaluating their proportions within specific spatial domains [136, 137]. Recent advances in cell-type annotation and deconvolution have produced a diverse array of computational tools, ranging from marker-based methods to sophisticated probabilistic and DL-based frameworks [138].

Reference-based methods transfer information from scRNA-seq atlases to spatial data. Cell2location estimates spatial cell-type abundances using a probabilistic model, CARD uses spatially informed regression, RCTD models mixtures of reference cell types, and DestVI represents both cell-type proportions and continuous within-type states [139–142]. For imaging-based data with cellular resolution, segmentation and annotation frameworks such as Bering may be more appropriate than spot-level deconvolution [143, 144]. The details of the cell-type annotation and deconvolution tools are given in Supplementary Table 5

Here, the deconvolution accuracy depends heavily on the quality of the scRNA-seq reference. Probabilistic frameworks (Cell2location, RCTD) outperform regression-based methods on heterogeneous tissues but are computationally heavier. CARD offers a balance of accuracy and speed via spatial smoothing. For imaging-based platforms with subcellular resolution, segmentation-first methods (Bering) are preferred. Reference-free approaches remain an open challenge.

#### Spatially variable genes detection

Spatially variable genes are genes that exhibit varying expression patterns across different regions within a sample [145]. Detection of SVGs is a crucial step in downstream analysis to understand tissue organization, cell–cell communication, and disease processes. Numerous computational methods, varying from statistical models to GNNs and ensemble frameworks, have been developed to identify SVGs [146, 147].

For instance, SPARK-X provides a scalable non-parametric framework, Moran’s I offers a transparent spatial-autocorrelation baseline, and methods such as STAMarker use learned spatial representations to identify domain-associated genes [148–150]. There are also cell type-specific SVG detection methods and classical spatial autocorrelation approaches to determine SVGs.

Similar to non-STs, differential expression results can be influenced by sequencing depth, in addition to tissue boundaries, cell-type composition, and the spatial domains used in the analysis. Importantly, a gene can therefore appear spatially variable because its expressing cell type is localized, rather than because it is spatially regulated within that cell type. Thus, findings should be confirmed and interpreted with cellular composition and tissue structure in mind. The list of all the tools for SVG detection are given in Supplementary Table 6.

#### Spatial interaction analysis

Spatial interaction analysis aims to identify relationships among neighboring cells, cell types, or genes. Gene–gene co-expression analysis can determine spatially correlated regulatory modules and molecular pathways [151], whereas cell–cell communication analysis via ligand-receptor signaling and proximity information could unveil how neighboring cell types interact within their microenvironment [152]. There are cell–cell communication tools, statistical-based methods, network-based methods, and ligand-receptor and spatial proximity frameworks (Supplementary Table 7). Gene–gene co-expression tools detect spatially structured gene networks, with recent advancements such as Smoothie, which uses Gaussian smoothing to infer genome-wide gene correlation networks from denoised ST data [153]. CellChat and NicheNet provide widely used non-spatial or reference frameworks for signaling inference, whereas SpatialDM and CytoSignal explicitly incorporate spatial organization [154–157].

These methods are used to identify genes with pairwise relationships that are spatially structured, thereby revealing regulatory networks that influence tissue phenotype. However, the predicted interactions should be regarded as hypotheses that need to be validated across samples and methods. Methods that combine ligand-receptor evidence with explicit spatial proximity are stronger, but conclusions can still depend on segmentation and deconvolution. The tools for spatial interaction analysis are enlisted in Supplementary Table 7.

#### Spatial trajectory and temporal analysis

Spatial trajectory and temporal analysis in ST offer insights into the dynamic changes and movements of cells within their native environments over time. It aids in reconstructing dynamic cellular processes, such as differentiation, development, and disease progression [158]. These analyses integrate the physical location of each cell (spatial coordinates) with its gene expression data, as well as the speed and direction of changes [159]. It helps determine the order in which cells transform, how they are related, interact with each other, and evolve over time. The combination of spatial and temporal information provides deeper insights into developmental and disease processes that conventional single-cell RNA-seq methods cannot capture [160, 161]. spVelo combines expression modeling with graph-based spatial information, Cell2fate models transcriptional dynamics through RNA-velocity modules, and SpaTrack uses optimal transport to infer spatial cell-state transitions [162–164]. Most ST datasets provide static observations rather than direct lineage measurements and spatial proximity is not equivalent to actual temporal succession. Thus, application of trajectory analysis should be employed only when the biological system supports the ordering process. The tools used for spatial trajectory and temporal analysis are enlisted in Supplementary Table 8.

#### Integration with multi-omics and histology data

Integrating ST data with other omics data, such as proteomics, epigenomics, and image-omics (detailed tissue image readings), provides a deeper biological insight into tissues and diseases [165, 166]. This multimodal approach enables a more comprehensive understanding of biological systems, encompassing cellular composition, spatial organization, and molecular interactions within tissues. However, integrating multimodal data presents several challenges, including data heterogeneity, batch effects, and missing values [123]. Employing hypergraph and contrastive learning, SpatialEx facilitates the integration of multiple omics layers guided by histology, expanding molecular profiling beyond single-omics assays [167]. ResST, a self-supervised learning model based on GNNs and margin disparity discrepancy, integrates gene expression, spatial locations, and tissue morphology data to identify spatial domains across the datasets while correcting for batch effects [168]. NicheCompass is a graph-based DL approach that models cellular interactions and characterizes cell niches by integrating spatial multi-omics data at cell- and spot-level resolution [169]. BANKSY is a ML-based framework that utilizes a spatial clustering algorithm to integrate spatial and molecular features through neighborhood transcriptomes and their microenvironment, supporting batch correction and scalable clustering [170]. MOFA+ is a statistical framework and a classical baseline that allows the integration of multi-omics modalities, including single-cell data [171]. Overall, the integration of ST data with multi-omics and histology data enables high-resolution analyses that unveil cellular interactions and tissue architecture, which are essential for biological research and translational medicine. The details of the tools are given in Supplementary Table 9.

Integration is complicated by differences in spatial resolution, feature sets, noise, missing modalities, and sample correspondence. Thus, starting such integrative analyses with matched-cell or matched-section samples is a sound approach when possible. Further, the integrated analyses should be compared with unimodal baselines and evaluated for modality-specific signal and biological interpretability, and out-of-sample robustness. Added algorithmic complexity should be justified by improved accuracy, interpretability, or discovery.

#### 3D spatial reconstruction

3D spatial reconstruction enables creation of a high-resolution 3D model of tissues by combining multiple layers of 2D tissue sections. The reconstructed 3D model can be analyzed for SVGs, signaling domains, or interaction networks in 3D space. Recent computational tools combine image registration, DL, and graph-based methods to perform slice alignment, batch correction, and 3D domain identification, facilitating studies of TME, developmental processes, and neural circuits [172–174]. So3D is a comprehensive 3D spatial omics database containing 72 high-quality 3D spatial transcriptome datasets across four species. It also provides interactive visualization and analysis modules for 3D spatial domain identification, cell-type mapping, and the detection of differential gene expression [173]. Open-ST is a framework that provides tools for efficiently capturing and analyzing transcripts in 2D and 3D spaces at subcellular resolution [172]. Pyxa (a platform by Stellaromics) enables 3D ST of thick, intact tissues using STARmap technology [63]. X-Pression is a cost-effective method that employs deep CNNs to reconstruct 3D gene expression signatures from microcomputed tomography data [175]. isoST uses AI to reconstruct 3D ST profiles with isotropic resolution from sparsely sampled serial sections [176]. SpaBatch is a DL-based framework developed for cross-slice integration and 3D reconstruction. It performs embedding learning, batch correction, and clustering optimization simultaneously for accurate spatial domain identification [177]. Deep-STARmap and Deep-RIBOmap can quantify thousands of transcripts and their translation activities within 200-μm intact thick tissue sections. These tools also facilitate 3D spatial profiling, enabling quantitative analysis of tumor-immune interactions in human skin cancer [174]. Although tissue deformation, missing sections, unequal spacing, batch effects, and limited ground truth can complicate evaluation, these methods uncover hidden patterns in 2D images of tissues by transforming them into 3D reconstructions, thereby enhancing our understanding of tissue architecture and disease mechanisms. Details of these tools are presented in Supplementary Table 10.

#### Advanced visualization

Visualization is essential for analyzing ST data, as it transforms complex, high-dimensional spatial gene expression data into clear and interpretable visual representations. Effective visualization methods help in studying tissue architecture, interpreting cellular interactions and organization, exploring spatial gene expression patterns, and identifying spatial domains. Various advanced visualization tools have been developed to help researchers interpret complex data more effectively. These include SCNT, a user-friendly R package that offers high-resolution spatial visualization along with customizable gene expression and clustering plots [178]. spatialGE is a web application that facilitates interactive data visualization for quality control, domain detection, and spatial gene expression exploration [179]. Vitessce is another web-based platform for interactive visualization of multi-modal and spatial data [180]. Napari is a Python-based interactive image viewer designed for browsing, annotating, and analyzing large datasets of multi-dimensional images [106]. TissUUmaps can be used to visualize gene expression data on top of whole slide images [181]. Some platform-specific viewers can support initial quality control but may offer limited reproducibility or cross-platform interoperability [182]. Since visualization choices can substantially affect data interpretation, interactive exploration should therefore complement rather than replace quantitative testing. Visualization tools should be selected according to dataset scale, image support, multi-modal capability, and interoperability. Using consistent color/number scales when comparing samples is also essential. Additional platforms that provide insightful visualizations are given in Supplementary Table 11.

Across these downstream analysis tasks, AI contributes most clearly where spatial, image, graph, or multi-modal relationships cannot be represented adequately by conventional (non-spatial) methods. CNNs support image analysis, GNNs model tissue neighborhoods, autoencoders learn nonlinear representations, and multi-modal models connect gene expression with morphology or other molecular layers. However, model selection should be supported by appropriate baselines, sensitivity analyses, uncertainty assessment, and possibly benchmark evidence or external validation. Systematic comparisons show that performance is conditional on the platform, tissue, pre-processing workflow, reference data, ground truth, and evaluation metric [131, 137, 145, 183–186]. No single method consistently dominates across all ST tasks or datasets. AI tools should therefore be selected as part of an evidence-guided workflow rather than according to novelty alone, with measured, inferred, and imputed quantities clearly distinguished throughout the analysis.

## Applications of ST

ST has transformed molecular biology by mapping gene expression within its spatial context, linking molecular signatures to tissue architecture [187–189]. Its applications span cancer biology, neuroscience, immunology, developmental biology, infectious disease, drug discovery, and plant biology (Fig. 2). By preserving spatial information, ST enables researchers to study not only which genes are active but also the specific locations where they are active within tissues, offering an extensive view of cellular interactions, microenvironmental cues, and disease mechanisms. These insights support advances in biomarker discovery, therapeutic targeting, and diagnostics.

### Cancer and tumor microenvironment

The spatially resolved information from ST reveals patterns of heterogeneity, cellular interactions, and organization niches that bulk and conventional single-cell RNA-seq cannot capture. Hence, its applications extend from basic tumor biology to biomarker discovery and therapy response prediction [190, 191]. One of the significant applications of ST is to characterize the TME, including immune, stromal, and vascular compartments and their spatial relationships to tumor cells [192]. By detailed mapping, ST defines the spatial distribution and functional states of various immune cells, including T cells, B cells, myeloid cells, fibroblasts, and endothelial cells. It helps in identifying immunosuppressive regions, cancer-associated fibroblast phenotypes, and tertiary lymphoid structures, all of which play a role in initiating antitumor responses [193–195]. The spatially resolved maps of the TME can guide the design of immunotherapies and rational combination of treatments aimed at targeting specific niches, rather than treating the tumor as a uniform mass [196].

Another major ST application in cancer is to define spatial gene expression signatures associated with prognosis, metastasis risk, and treatment response [197]. By integrating ligand-receptor analysis with spatial proximity, ST could reveal specific cell–cell communication axes that drive tumor progression, angiogenesis, and immune suppression [192, 196]. ST is also used to examine invasive margins, pre-metastatic niches, and metastatic lesions to understand local invasion and distant spread [198, 199]. Profiling of primary tumors, surrounding tissue, and metastasis sites has revealed specific gene expression changes that facilitate colonization. These changes include altered fibroblast states and immune exclusion at metastatic sites [200, 201]. This helps us understand how tumors alter local environments during progression and suggests therapies targeting specific areas to prevent or treat metastasis.

In drug discovery and translational oncology, ST is utilized to assess how candidate therapies influence the spatial patterns of gene expression and cell states within the TME, enabling the identification of on-target effects, resistance mechanisms, and off-target effects [187, 202, 203]. Spatial profiling before and after treatment can help identify the emergence of resistant clones and highlight where protective microenvironments persist. This information can guide rational drug combinations, optimize doses, and support development of spatially informed biomarkers for improved clinical decision-making.

### Neuroscience

ST offers valuable neurological insights by resolving the complex cellular organization of the brain while preserving anatomical context [204]. By generating high-resolution gene expression atlases across distinct brain regions, possibly over time, ST applications enabled a more precise study of brain development, normal physiology, and disease mechanisms [205]. In neurodegenerative diseases, such as Alzheimer’s disease (AD), Parkinson’s disease, and ALS, ST has been used to examine dysregulated gene networks and transcriptional changes occurring in the structural tissue domain surrounding amyloid plaques [24]. Spatial analyses in AD identified distinct subpopulations of microglia and astrocytes that cluster around plaques. These subpopulations exhibit unique inflammatory and disease-associated transcriptional signatures, revealing localized cellular networks that contribute to neuroinflammation and synaptic dysfunction [206]. In ALS, it maps region-specific changes in glia and neurons within the spinal cord and brainstem, enhancing the understanding of how degeneration occurs over time and in different areas [207].

ST has also provided valuable insights into neuropsychiatric disorders, stroke, and epilepsy. It can help detect changes in gene expression and cell states that occur in specific cortical layers, hippocampal subfields, or other vulnerable regions following injury or in disease models [205]. These studies showed spatially localized changes in synaptic, inflammatory, and metabolic pathways, along with the reorganization of both inhibitory and excitatory circuits, which cannot be detected through bulk profiling [204, 206]. By integrating ST with electrophysiology, imaging, and behavioral data, researchers can directly link molecular pathology to functional circuits and phenotypes in these conditions [208].

In addition to its role in studying diseased conditions, ST also contributes to study brain development and circuit assembly. It can map spatiotemporal gene expression patterns that direct neurogenesis, migration, and lamination in both the embryonic and postnatal brain [204, 209]. ST can help outline the development of neuronal and glial lineages, identify regional patterning cues and niche factors that influence the formation of functional circuits, offering mechanistic insight into how early developmental perturbations may predispose to later neurological or psychiatric diseases [189, 204]. These applications position ST as a pivotal integrative tool in neuroscience, linking molecular states, spatial architecture, and circuit function in both healthy and diseased conditions.

### Immunology and developmental biology

ST has transformed immunology by enabling researchers to study immune cells within their native tissue context. This approach reveals how the localization and functional states of these cells are influenced by the surrounding cells and extracellular matrix [210, 211]. Unlike traditional transcriptomics, which dissociate tissues and lose spatial information, ST maps immune cells within their native tissue environments, revealing how location shapes function in organs such as lymph nodes, the spleen, the liver, the intestine, and tumors [169]. It identifies specialized immune niches where B cells, T cells, dendritic cells, or macrophages adopt distinct transcriptional states based on their spatial context [210].

In developmental biology, ST captures spatiotemporal gene expression patterns during embryogenesis, organogenesis, and tissue patterning, while preserving the 3D context that is typically lost in dissociated samples [212, 213]. By profiling whole embryos at high resolution, ST can help reconstruct molecular gradients, cellular trajectories, and signaling centers that drive morphogenesis, revealing how transcription factors, enhancers, and chromatin states vary across tissues and developmental stages [214, 215]. ST is also used to study remodeling and regeneration in organs such as the heart, kidney, liver, and musculoskeletal tissues, revealing cell signaling networks supporting wound healing and tissue regeneration [216].

### Infectious diseases

ST is transforming infectious disease research by simultaneously mapping the gene expression of both pathogens and hosts within their native tissue context. This reveals the localization of viruses, bacteria, or parasites and highlights how host responses vary in different spatial regions [217, 218]. In contrast to conventional methods, ST illustrates how infection disperses through organs, identifies microenvironments where immune responses succeed or fail, and reveals patterns of local tissue damage that clarify clinical pathology [219, 220].

In studying viral infections, ST has been extensively used to analyze SARS-CoV-2, where it maps the distribution of viral RNA in the lungs, brain, and other organs, while also identifying host immune signatures [219, 221, 222]. Research has shown that infected cells tend to cluster in specific areas, such as alveolar regions, bronchiolar epithelium, and endothelial cells. These findings reveal diverse interferon responses and macrophage activation, which correlate with disease severity and the neuropathological complications associated with long COVID [223]. Similar approaches have been used for influenza, HIV, and herpesviruses to study tropism, immune evasion niches, and persistent reservoirs [224].

In bacterial infections like tuberculosis, ST is used to analyze the architecture of granulomas. It can reveal necrotic cores surrounded by specific immune layers, each characterized by distinct states of macrophages, T cells, and fibroblasts, hence explaining drug resistance and the persistence of the infection [225]. By providing spatially resolved insights, ST identifies potential therapeutic targets in the areas where the infection is most active, as well as locations where the immune response is failing across viral, bacterial, and parasitic infections. By integrating ST with single-cell resolution imaging, researchers can study the intricate interactions between pathogen dissemination, immune response, and collateral tissue damage that influence infection outcomes [226].

### Drug discovery and precision medicine

ST is being investigated in drug-discovery studies to localize treatment-associated transcriptional changes, identify spatially restricted disease pathways, and characterize resistant or treatment-responsive tissue regions. Current applications include target prioritization, analysis of treatment mechanisms, toxicity assessment, and evaluation of candidate spatial biomarkers. Most such applications remain preclinical or retrospective [88, 187]. In contrast to conventional transcriptomic approaches, which often overlook anatomical context, ST reveals the precise locations where drugs modulate gene programs. ST can highlight which cell types respond to treatment and which niches remain resistant. It supports precision medicine by enabling spatially informed biomarker assessment and patient stratification, helping predict treatment response and resistance in clinical and translational studies [227].

In target discovery, ST identifies novel drug targets by highlighting SVGs enriched in disease-specific regions, such as tumor invasive fronts or inflamed tissue zones [187]. It links these genes to druggable pathways, facilitating drug repurposing, such as identifying tumor specific antigens to trigger monocyte-to-macrophage differentiation [228], as well as prioritizing drug candidates through platforms like SOAR, which integrate ST with protein interaction networks [88].

Drug mechanism and resistance profiling represent another key application where ST maps pre- and post-treatment tissues to visualize on-target effects, adaptive resistance mechanisms, and off-target toxicities *in situ* [120, 209]. In oncology, researchers utilize ST to monitor how therapies alter TME, identifying where resistant clones emerge, such as in hypoxic cores, or where protective stromal niches persist, aiding in drug combination strategies and dose optimization [37, 191].

ST can play a crucial role in developing biomarkers by identifying spatial gene signatures that can predict treatment responses and prognosis [229]. For complex diseases such as cancer, it could map drug targets across diverse tissue types, guiding combination therapies and assessing the likelihood of treatment resistance. ST is positioned to be integrated into clinical workflows for companion diagnostics, digital pathology, and personalized treatment planning, bridging molecular insights with histopathological phenotypes [187, 227].

### Plant biology

In plant biology, ST is transformative, by overcoming longstanding challenges posed by plant cell walls, large vacuoles, and complex tissue architectures that complicate traditional single-cell approaches [230]. Recent advances in platforms such as Stereo-seq, Slide-seq, and MERFISH have enabled high-resolution mapping of gene expression in plant tissues. This has revealed insights into cellular diversity, developmental pathways, and responses to stress, all while preserving the spatial context of the samples [28, 231]. In stress and environmental biology, ST examines how gene regulatory networks are activated in specific tissues or cell types under conditions such as drought, salt stress, pathogen attacks, and nutrient deficiencies [230]. It also explores wound responses and symbiotic interactions. It can reveal how different cell types (epidermal, cortical, or endodermal) contribute distinct transcriptional responses to both mutualistic and pathogenic relationships [27, 28].

Finally, crop improvement and functional genomics applications leverage ST to identify trait-associated regulatory hubs and breeding targets [232]. In crops such as maize, rice, and tomato, ST has mapped processes including kernel development, grain filling, and fruit ripening, and revealed spatial regulators that influence yield, nutritional content, and stress tolerance [27]. By incorporating ST with genome-wide association studies and CRISPR screenings, researchers can identify non-coding enhancers and cell-type-specific promoters that drive important agronomic traits. Future integration with spatial proteomics and metabolomics could also lead to comprehensive multi-omics atlases, enhancing the precision of breeding and bioengineering to develop climate-resilient crops [230, 233].

### Towards translational studies

To illustrate the translational impact of ST, below we highlight three examples where ST data involves clinical samples or studies that could potentially inform clinical decision-making.

He et al. [229] integrated ST gene expression from primary breast tumors with matched histological images using a deep learning framework. In addition to accurately predicting gene expression, the method captured biologically relevant image features, separating tumor from normal tissue and flagging incongruent annotations. Notably, the work demonstrates the potential to facilitate novel image-based screening approaches for molecular biomarkers exhibiting spatial heterogeneity, thereby creating promising opportunities for new clinical applications and use of DL tools to aid identify subtle biological patterns.

Chen et al. [24] applied ST to AD mouse model brain sections, identifying gene clusters specifically regulated by amyloid plaque depositions, possibly not detectable in bulk or dissociated single-cell analyses. Their findings could implicate adjustments to evaluating drugs and antibodies that target amyloid plaques. Such preclinical work shows how ST incorporation can improve mechanistic and therapeutic hypothesis generation.

Cross et al. [219] applied ST to lung tissue from COVID-19 patients at the point of death and identified spatially structured immune circuits, namely a coordinated immune-cell signaling network in severely affected tissue, driven by interactions between cytotoxic lymphocytes and pro-inflammatory macrophages. With their network-based analysis and mechanistic insights, the authors showed that combination therapies, drugs directly targeting the virus and those tempering the dysregulated host immune responses, could be better at handling heterogeneity of the tissue pathology. The work illustrated how ST use can help mechanistic characterization of the severe disease.

Abovementioned studies demonstrate the translational potential of ST but progress toward clinical use would require prospective cohorts, standardized (FFPE) workflows, predefined endpoints, external validation, and demonstration of value over conventional pathology to alter diagnosis, prognosis, or treatment selection.

## Role of AI in ST

AI plays a multifaceted role in ST by transforming raw, high-dimensional data into actionable biological and clinical insights. AI automates important preprocessing tasks, such as tissue segmentation and spot-level deconvolution. For tissue segmentation, DL models such as U-Net variants or StarDist are used to delineate cellular boundaries from H&E stains. In terms of spot-level deconvolution, methods such as Tangram, Spatial-ID, and Cell2location help map scRNA-seq references to larger ST spots to infer cell-type proportions [234]. In addition to data preprocessing, tools such as SpaGCN and STAGATE use GNNs to identify spatial domains. They represent different regions of the tissue as nodes in a graph, enabling the modeling of complex, non-Euclidean relationships, allowing the identification of functionally distinct areas, such as tumor cores, invasive fronts, and immune niches [235]. Transformers and VAEs enhance SVG detection and trajectory inference by learning latent representations that emphasize genes with coordinated, location-dependent expression patterns. A key strength of AI-foundation models is their ability to integrate across different modalities. For example, pre-trained models like PLIP [236], BiomedCLIP [237], OpenAICLIP [238], and CONCH [239] effectively align ST with imaging and omics data [240].

AI can drive clinical translation by bridging spatial signatures to patient outcomes. Some studies have associated spatial expression patterns with prognosis, treatment response, or disease classification, indicating that AI-based analysis may support the development of spatial biomarkers and patient-stratification models. However, most models have been evaluated retrospectively and require external, prospective, and clinically relevant validation before they can support decision-making. AI transforms ST from simple descriptive profiling into predictive and interactive systems, making complex analyses easier to understand for both biologists and clinicians.

Despite the proliferation of AI tools, systematic head-to-head benchmarking remains limited. Table 5 summarizes the scope, principal findings, practical implications, and limitations of these evaluations. For spatial clustering, the most comprehensive benchmark to date evaluated 19 methods (14 of which are recent and GNN based) on 57 datasets, finding that STAGATE and GraphST consistently outperformed classical clustering methods, though no single method dominated across all [131]. For cell-type deconvolution, Li et al. [137] benchmarked 18 tools, finding that Cell2location and CARD achieved the best balance of accuracy and robustness, while faster regression-based methods (SPOTlight, SpatialDWLS) sacrificed accuracy on spot-heterogeneous tissues. For SVG detection, Li et al. [145] benchmarked 14 methods and found SPARK-X [148, 241] as top-performing method in terms of sensitivity and specificity, performances generally depending on model assumptions, technology, and tissue type.

**Table 5:** Comparison of benchmark results for major spatial transcriptomics analysis tasks.

Analysis task	Benchmark study and scope	Evaluation metrics	Main benchmark result	Practical recommendation	Important limitations
Spatial-domain identification	Kang et al. [131]: 19 methods, including 14 recent deep-learning methods; 30 real datasets spanning six ST technologies and 27 synthetic datasets.	Accuracy, stability, generalizability, scalability, and effects of domain identification on downstream SVG detection.	No method performed best across all datasets. Performance depended strongly on the ST platform, but STAGATE and GraphST are among the consistently strong methods. More accurate domain identification generally improved the number and accuracy of domain-specific SVGs, but outputs from different GNN methods showed low concordance.	Select methods according to platform, resolution, tissue architecture, and computational scale rather than assuming that a particular GNN is universally superior. Use a spatial method together with a non-spatial baseline.	Ground-truth domains in real tissues are imperfect, and benchmark rankings depend on metrics, annotations, pre-processing, graph construction, and the platform composition of the benchmark. GNN performance for domain identification depends on how spatial graphs and feature embeddings are constructed, which varies significantly between sequencing and imaging platforms.
Cell-type deconvolution	Li et al. [137]: 18 methods evaluated across 50 real and simulated datasets, varying ST technology, resolution, spot number, gene number, and evaluation metric.	Accuracy, robustness, usability, computational efficiency, and sensitivity to data characteristics.	CARD, Cell2location, and Tangram achieved the strongest overall performance in the benchmark. The study nevertheless recommended scenario-specific selection rather than a single universal method.	Use CARD or Cell2location as benchmark-supported starting points for spot-level composition estimation; consider Tangram when mapping single-cell profiles to spatial locations is central.	Results depend on reference-cell composition, rare-cell representation, platform mismatch, and the accuracy of ground truth. The review predates several recently developed methods. Also, subcellular/single-cell imaging platforms bypass deconvolution in favor of cell segmentation and cell typing.
Spatially variable gene detection	Li et al. [145]: 14 methods evaluated using 96 spatial datasets and six metrics, including extension to spatial ATAC-seq.	Sensitivity, gene ranking, classification, statistical calibration, computational scalability, downstream usefulness, and extendibility.	SPARK-X showed the strongest overall performance, while Moran’s I was a competitive and interpretable baseline. Most evaluated methods showed poor statistical calibration, and methods developed for SVGs did not necessarily transfer effectively to spatially variable peak detection.	Use SPARK-X as a benchmark-supported starting point and retain Moran’s I as a transparent baseline. Validate important SVGs with a second method based on different assumptions and assess confounding by cell-type composition.	Performance varies with spatial pattern, sequencing depth, gene prevalence, platform, and the balance between simulated and real datasets. Note that Moran’s I represents a standard statistical baseline rather than a standalone ST pipeline.
	Chen et al. [183]: benchmark framework using 30 synthetic and 74 real-world datasets.	Accuracy across synthetic and biological datasets, application scenarios, and method-selection guidance.	Further demonstrated that SVG-method performance is scenario-dependent, supporting the need to select methods according to data characteristics rather than applying a universal ranking.	Record and report data characteristics (platform, resolution, gene-panel, spatial pattern, and sample size) when justifying SVG-method selection. Agreement between independently developed benchmarks should be treated as stronger evidence than rankings from a single study.	Differences in simulation design, ground-truth definition, filtering, and evaluation metrics can produce different rankings between benchmark studies.
3D reconstruction and multi-slice integration	Yan et al. [184] and Dong et al. [185]: Recent evaluations compare alignment and multi-slice integration using datasets with varying registration, platform, and tissue characteristics.	Alignment accuracy, biological conservation, scalability, cross-slice integration, and downstream utility.	Indicates that the best alignment or integration method is dataset- and objective-dependent; a method that performs well for anatomical registration may not be optimal for batch correction, domain preservation, or downstream analysis.	Define the primary objective before selecting a method: geometric registration, molecular integration, batch correction, domain correspondence, or 3D reconstruction. Evaluate geometric and biological performance separately.	Ground truth is often unavailable for real serial tissue sections. Tissue deformation, missing regions, biological changes between sections, and conflicting evaluation metrics complicate method ranking.
LLM-assisted ST interpretation	Current evidence is dominated by individual framework papers and small demonstrations; a mature independent benchmark covering LLM-based ST tools is not yet available.	Natural-language querying, annotation, knowledge integration, niche identification, interpretability, and biological plausibility.	No LLM-based ST method can yet be identified as a field-wide benchmark winner. Current systems are promising for exploratory interpretation but are not sufficiently validated to replace statistical or task-specific computational methods.	Restrict LLMs to literature-assisted annotation, structured querying, report generation, and hypothesis generation after quantitative analysis. Require traceable links between generated statements and genes, regions, statistical outputs, and supporting literature.	Hallucination, prompt sensitivity, data leakage, uncertain knowledge provenance, limited cross-platform testing, and absence of prospective validation remain major obstacles.

Collectively, these benchmarks demonstrate that no single computational method consistently outperforms all alternatives across datasets and analytical objectives. Graph-based models perform strongly for spatial-domain identification, whereas probabilistic and spatially regularized approaches are competitive for cell-type deconvolution. However, spatial-domain performance depends substantially on the experimental platform and tissue architecture, while deconvolution accuracy is strongly influenced by the completeness and compatibility of the single-cell reference. Method rankings may also vary with preprocessing, ground-truth definition, simulation design, evaluation metric, and computational scale.

As summarized in Fig. 3, AI-method selection should therefore be guided by the analytical task, data-generating platform, availability of reference data and complementary modalities, computational requirements, and desired level of interpretability. Where feasible, users should compare at least two suitable method classes, inspect their spatial outputs, and assess the stability of key conclusions under alternative preprocessing choices and parameter settings. Uncertainty assessment and independent biological validation are particularly important for findings central to the study. Comprehensive inventories of tools and methods are provided in Supplementary Tables 1–11.

### Limitations of current AI methods

Although AI methods can extract complex spatial and multimodal patterns from ST datasets, the performance is still dependent on the tissue, platform, pre-processing workflows, and reference data. For instance, models trained predominantly on Visium or a restricted set of tissues may not transfer reliably to imaging-based, subcellular, 3D, or clinically archived datasets. So, geographical or chronological differences in tissue preparation, staining, gene panels, and imaging can therefore produce substantial performance degradation that is not apparent from within-dataset validation.

There are also task-specific failure modes. GNNs may oversmooth sharp biological boundaries or propagate segmentation and neighborhood-construction errors. Batch-correction and integration models may remove genuine biological variation. Deconvolution methods inherit biases from incomplete or mismatched scRNA-seq references and may fail to resolve rare, transitional, or disease-specific cell states.

Scalability and reproducibility remain additional concerns. Many methods are evaluated on relatively small, curated datasets and may require substantial memory, GPU resources, or platform-specific parameter tuning when applied to millions of cells or terabyte-scale images. Comparisons are further complicated by inconsistent pre-processing, metrics, ground truths, software versions, and train-test splits. Recent benchmarking work similarly indicates that optimal method performance is frequently dataset-dependent rather than universal [184, 185].

LLM-based systems introduce further risks, including hallucinated biological interpretations, prompt sensitivity, uncertain evidence provenance, and limited independent validation. They should therefore be used primarily for exploratory querying, annotation support, and hypothesis generation after conventional statistical analysis, rather than as standalone analytical or clinical decision systems. Reliable deployment will require externally validated benchmarks, uncertainty quantification, data-provenance safeguards, interpretable outputs, and prospective testing across platforms, institutions, and patient cohorts.

## Role of large language models in ST

The AI tools described above (GNN-based or VAE-driven) share a common paradigm that they are trained discriminatively on spatial omics data to perform specific analytical tasks. LLMs represent a fundamentally different paradigm: they are generative, trained on vast text corpora, and are applied to ST not to replace existing tools but to provide a natural-language interface, feature encoding, and automated biological interpretation layered on top of statistical outputs. Therefore, LLMs are emerging as an interpretive and integrative layer above the analytical stack, not as a replacement for the ML and DL methods described above. These models simplify the interpretation of high-dimensional data and link gene expression patterns with biological processes [242]. LLMs can effectively translate complex outputs, such as spatially resolved gene modules, pathway enrichments, and cell–cell interaction scores, into coherent textual explanations that are easier for researchers and clinicians to interpret. Recent frameworks, such as QuST-LLM, demonstrate how LLMs can be integrated into established ST workflows. This integration provides bidirectional querying, summarizing the biology of selected tissue regions and retrieving regions that match user descriptions of microenvironments or processes [243]. In addition to text summarization, LLMs are being utilized as knowledge-infused feature encoders. Methods such as spaLLM create embeddings for genes and cells by incorporating textual knowledge (gene names, functions, and literature context) and merging this information with spatial neighborhood data to enhance spatial domain identification, particularly in multi-omics or sparse datasets [244]. LLMiniST presents a new zero-shot approach for identifying spatial niches. This method transforms ST data into context-aware prompts that include information about neighboring gene expression, cell type composition, tissue metadata, and external knowledge, outperforming traditional clustering methods and offering enhanced generalizability through a two-stage fine-tuning process [245].

TISSUENARRATOR represents tissue sections as spatial sentences, using ranked gene lists that are enhanced with coordinates and metadata. This allows pretrained language models to create realistic cellular profiles, predict interactions, conduct in silico perturbations, and facilitate natural-language queries across technologies such as MERFISH and CosMx [246]. PGL is another LLM-based framework that reinterprets single-cell transcriptomes as a language generation problem. It uses LLMs to create comprehensive scRNA-seq profiles from metadata, such as tissue type and disease context. The generated cell profiles serve as effective references for ST, allowing for accurate cell-type mapping without the need for matched single-cell atlases. PGL facilitates the construction of virtual cohorts, aids in hypothesis generation, and supports on-demand reference analysis, positioning generative language models as essential tools for in silico single-cell biology [247]. spEMO is a computational framework that integrates embeddings from pathology foundation models and LLMs to analyze spatial multi-omics data. It surpasses single-modality models in various downstream tasks, including spatial domain identification, spot-type classification, disease prediction, multicellular interaction inference, and automated medical reporting. Additionally, spEMO has introduced a novel multi-modal alignment benchmark to assess how effectively pathology foundation models retrieve complementary information [248].

Recent reviews of AI in transcriptomics highlight that LLM-derived embeddings can reduce the requirement for handcrafted feature engineering and facilitate the integration of various spatial modalities, such as RNA, proteins, and morphology [249]. Multi-modal foundation models further enhance research by integrating gene expression, histology images, and textual annotations into shared representations. This enables LLMs as interactive systems where researchers can pose natural-language questions about spatial patterns and receive responses that include both spatial selections and textual explanations [248].

While LLMs offer promising interpretive capabilities in ST, several critical limitations temper their widespread adoption. First, most LLM-based tools (QuST-LLM, spaLLM, LLMiniST, and TISSUENARRATOR) have been benchmarked on a small number of datasets, often only MERFISH or CosMx data, limiting generalizability across platforms and tissue types. Independent validation on prospective or clinical cohorts is absent. Second, LLMs are prone to hallucination, generating biologically plausible but factually unsupported interpretations of gene modules or pathway enrichments without providing traceable evidence back to the underlying data. Third, the integration of LLMs with existing statistical pipelines (normalization, deconvolution, and clustering) is still largely ad hoc; no standardized API or benchmarking framework exists to evaluate LLM-ST tool performance against ground truth. Fourth, the computational and financial costs of running large-scale LLMs on full spatial datasets are non-trivial, creating accessibility barriers for many research groups. Finally, most current LLMs are general-purpose and have not been trained on ST-specific biological literature or curated spatial atlases, meaning domain-specific performance remains inferior to dedicated statistical or DL models for core tasks. Until prospective validation studies and standardized benchmarks are established, LLM tools in ST are best viewed as hypothesis-generating assistants rather than reliable analytical engines.

## Challenges and limitations of ST

Despite rapid technological advancements, ST faces significant technical, computational, analytical, and translational limitations. One of the key challenges in ST is the technical limitations of the current platforms and their trade-offs between resolution and coverage. For instance, conventional 10× Visium provides whole-transcriptome profiling at 55 μm spot resolution, with each spot potentially containing transcripts from multiple cells. Visium HD increases the density of spatial barcodes to 2 μm, although gene-expression values are commonly aggregated into larger bins to improve sensitivity and interpretability. In contrast, imaging-based platforms such as CosMx, Xenium, and MERFISH provide single-cell or subcellular localization but generally measure predefined gene panels rather than whole transcriptome. High-resolution sequencing platforms such as Stereo-seq and Seq-Scope provide broad gene coverage at fine spatial granularity, but their data can be sparse and computationally demanding. Thus far, no single platform has managed to provide unbiased whole-transcriptome data at true single-cell resolution across large tissue areas [115, 216, 250, 251]. Computational approaches have been proposed to address the persistent resolution-coverage trade-off in ST. For example, whole-transcriptome profiles may be inferred from targeted imaging panels using paired scRNA-seq references. However, these imputation strategies introduce additional uncertainty and have not yet been validated for routine clinical applications [115, 250].

Additionally, most of the platforms exhibit low capture efficiency, particularly for low-abundance mRNAs, non-coding RNAs, and nuclear transcripts. This low efficiency can lead to dropout bias and reduced sensitivity in the data. Sample preparation also poses further challenges. Fresh-frozen samples yield the best results; however, FFPE clinical samples are susceptible to RNA degradation and crosslinking artifacts, which can compromise data quality. Thick or fibrous tissues such as those found in brain, muscle, or plants, present challenges for uniform sectioning and permeabilization [252–254].

Another major challenge is data processing and computational limitations. ST datasets produce large volumes of high-dimensional data, often reaching gigabytes-to-terabytes per tissue sample. Such data include raw images, sequencing reads, and spatial expression matrices, demanding storage systems, GPU clusters, and fast processors. Additionally, these datasets require specialized compression algorithms, as standard transcriptomics tools are not effective at managing spatial coordinates. Batch effects from different platforms, tissues, or labs complicate integration, and the lack of standardization hinders benchmarking and reproducibility [255]. A fundamental unresolved challenge is the absence of a universal data standard for ST. Formats differ across platforms (e.g. Visium’s HDF5 output, Xenium’s Parquet files, and MERSCOPE’s CSV-based transcripts), and metadata schemas are inconsistent, hindering FAIR data sharing and multi-cohort meta-analyses [96]. While initiatives such as the SpatialData framework and the SODB repository are working toward interoperability, widespread adoption remains limited. Compounding this, batch effects across ST platforms are more complex than in scRNA-seq because they involve spatial coordinate systems, image artifacts, and tissue-specific permeabilization efficiencies simultaneously. Benchmark studies have shown that the relative performance of batch correction tools varies drastically depending on tissue type and platform [121].

As ST moves to whole-organ and multi-section datasets, computational demands become prohibitive. Terabyte-scale datasets from Stereo-seq or Slide-seq are incompatible with standard single-machine workflows. Most existing tools were benchmarked on Visium datasets of ~3000–5000 spots; scaling to millions of cells per sample (e.g. Xenium HD) requires algorithmic redesign and GPU-accelerated implementations. Memory-efficient graph representations, approximate nearest-neighbor indices, and streaming data loaders are active research areas but not yet the default in most published tools.

Analytical challenges present additional difficulties, such as spot-level deconvolution using scRNA-seq reference results, which can lead to uncertain cell-type proportions in heterogeneous tissues. Emerging machine learning approaches have sought to address these analytical limitations by integrating STs with complementary data modalities. For instance, SpaIM uses scRNA-seq references to infer unmeasured spatial gene expression, whereas HGGEP predicts spatial gene expression directly from histopathology images using hypergraph neural networks and multi-scale morphological features [256, 257]. These methods have demonstrated improvements in gene-expression prediction accuracy and spatial domain characterization, suggesting that computational inference can partially compensate for current technical limitations of ST platforms. Nevertheless, their performance depends on training data quality, model assumptions, and biological context, raising concerns regarding generalizability, interpretability, and clinical applicability.

Furthermore, multi-omics integration encompassing techniques like spatial proteomics and epigenomics faces issues with mismatched resolutions, differing data types, and a lack of unified data formats, resulting in incompatible files. Additional obstacles include background noise correction from autofluorescence, handling variable resolutions, and scalable spatial domain detection without standardized benchmarks. When it comes to clinical translation, there are regulatory, cost, and validation challenges, along with a limited number of standardized workflows for FFPE samples and prospective studies [250, 253, 254].

## Future directions for ST

Looking ahead, the future of ST focuses on developing higher-resolution platforms, enhancing 3D reconstruction, and integrating multi-omics data. These advancements would aim to capture subcellular details and dynamic processes across samples from diverse individuals and conditions. AI holds immense potential to overcome critical challenges in ST, ranging from data processing to biological discovery [234]. Future developments will utilize DL models, including GNNs and variational autoencoders, to automate tasks such as segmentation and deconvolution. Future unified frameworks may use end-to-end multi-task learning with shared multimodal encoders to improve consistency across segmentation, representation learning, domain identification, and biological interpretation [33]. Foundation-model pretraining on large and diverse cross-platform cohorts may improve transfer to new tissues and technologies, while uncertainty quantification, spatial attention maps, interpretable latent factors, and counterfactual analyses could make model outputs more transparent. These developments should be evaluated through independent and prospective studies rather than assumed to confer zero-shot generalizability or clinical readiness [258]. These models will improve accuracy in analyzing complex tissues, enable scaling to terabyte-sized datasets, and facilitate the integration of multi-omics data, such as histology images, to create more streamlined analysis pipelines [249, 259].

AI-driven methods will reveal hidden spatial patterns, cell–cell interactions, and trajectory inference directly from raw ST data. This will support real-time mapping of TME and developmental processes, along with predictive models that forecast disease progression based on spatial gene gradients [260, 261]. AI enables more automated, robust, and interpretable pipelines that are needed for clinical deployment. AI-based models can segment cells, classify tissue regions, and associate spatial signatures with clinical outcomes or treatment responses, hence transforming complex ST data into clinically relevant scores and risk groups [234, 259]. AI is anticipated to transform raw, noisy ST data into standardized, interpretable, and clinically actionable formats, significantly reducing many technical and analytical constraints [262]. Explainable AI frameworks will enable personalized therapies through in silico screening, automate biomarker discovery, and address challenges related to reproducibility, regulatory compliance, and interpretability, ultimately bridging ST from being a specialized research tool to a resource for personalized diagnostics and decision-support applications.

**Key Points**

- Comprehensive review of the multifaceted role of AI in spatial transcriptomics, from preprocessing to clinical interpretation.
- Detailed overview of various ST technologies, key repositories, and major databases.
- In-depth discussion of AI-powered tools for ST data preprocessing and downstream analysis.
- This review also discusses the applications of ST in various domains as well as the role of LLMs in ST.
- Challenges, limitations, and future directions of ST have also been discussed.

## Supplementary Material

Supplementary_Tables_v02_bbag569

## Data Availability

Data sharing is not applicable to this article as no new data were created or analyzed in this study.

## References

[1] Larsson L, Frisén J, Lundeberg J. Spatially resolved transcriptomics adds a new dimension to genomics. Nat Methods 2021;18:15–8. 10.1038/s41592-020-01038-7 33408402

[2] Qin D . Next-generation sequencing and its clinical application. Cancer Biol Med 2019;16:4–10. 10.20892/j.issn.2095-3941.2018.0055 PMC6528456 31119042

[3] Day-Williams AG, Zeggini E. The effect of next-generation sequencing technology on complex trait research. Eur J Clin Investig 2011;41:561–7. 10.1111/j.1365-2362.2010.02437.x PMC3107422 21155765

[4] Tang F, Barbacioru C, Wang Y et al. mRNA-Seq whole-transcriptome analysis of a single cell. Nat Methods 2009;6:377–82. 10.1038/nmeth.1315 19349980

[5] Haque A, Engel J, Teichmann SA et al. A practical guide to single-cell RNA-sequencing for biomedical research and clinical applications. Genome Med 2017;9:75. 10.1186/s13073-017-0467-4 PMC5561556 28821273

[6] Li X, Wang C-Y. From bulk, single-cell to spatial RNA sequencing. Int J Oral Sci 2021;13:36. 10.1038/s41368-021-00146-0 PMC8593179 34782601

[7] Kharchenko PV . The triumphs and limitations of computational methods for scRNA-seq. Nat Methods 2021;18:723–32. 10.1038/s41592-021-01171-x 34155396

[8] Adema K, Schon MA, Nodine MD et al. Lost in space: what single-cell RNA sequencing cannot tell you. Trends Plant Sci 2024;29:1018–28. 10.1016/j.tplants.2024.03.010 38570278

[9] Crosetto N, Bienko M, van Oudenaarden A. Spatially resolved transcriptomics and beyond. Nat Rev Genet 2015;16:57–66. 10.1038/nrg3832 25446315

[10] Ståhl PL, Salmén F, Vickovic S et al. Visualization and analysis of gene expression in tissue sections by spatial transcriptomics. Science 2016;353:78–82. 10.1126/science.aaf2403 27365449

[11] Robles-Remacho A, Sanchez-Martin RM, Diaz-Mochon JJ. Spatial transcriptomics: emerging Technologies in Tissue Gene Expression Profiling. Anal Chem 2023;95:15450–60. 10.1021/acs.analchem.3c02029 PMC10603609 37814884

[12] Xu X, Su J, Zhu R et al. From morphology to single-cell molecules: high-resolution 3D histology in biomedicine. Mol Cancer 2025;24:63. 10.1186/s12943-025-02240-x PMC11874780 40033282

[13] Du J, Yang Y-C, An Z-J et al. Advances in spatial transcriptomics and related data analysis strategies. J Transl Med 2023;21:330. 10.1186/s12967-023-04150-2 PMC10193345 37202762

[14] Walker BL, Cang Z, Ren H et al. Deciphering tissue structure and function using spatial transcriptomics. Commun Biol 2022;5:220. 10.1038/s42003-022-03175-5 PMC8913632 35273328

[15] Pareja-Lorente E, Aloy P. Systematic assessment of microenvironment-dependent transcriptional patterns and intercellular communication. Genome Biol 2025;26:193. 10.1186/s13059-025-03677-5 PMC12232842 40619422

[16] Rao A, Barkley D, França GS et al. Exploring tissue architecture using spatial transcriptomics. Nature 2021;596:211–20. 10.1038/s41586-021-03634-9 PMC8475179 34381231

[17] Denisenko E, de Kock L, Tan A et al. Spatial transcriptomics reveals discrete tumour microenvironments and autocrine loops within ovarian cancer subclones. Nat Commun 2024;15:2860. 10.1038/s41467-024-47271-y PMC10991508 38570491

[18] Black S, Phillips D, Hickey JW et al. CODEX multiplexed tissue imaging with DNA-conjugated antibodies. Nat Protoc 2021;16:3802–35. 10.1038/s41596-021-00556-8 PMC8647621 34215862

[19] Lin J-R, Izar B, Wang S et al. Highly multiplexed immunofluorescence imaging of human tissues and tumors using t-CyCIF and conventional optical microscopes. eLife 2018;7:e31657. 10.7554/eLife.31657 PMC6075866 29993362

[20] Hinohara K, Polyak K. Intratumoral heterogeneity: more than just mutations. Trends Cell Biol 2019;29:569–79. 10.1016/j.tcb.2019.03.003 PMC6579620 30987806

[21] Berglund E, Maaskola J, Schultz N et al. Spatial maps of prostate cancer transcriptomes reveal an unexplored landscape of heterogeneity. Nat Commun 2018;9:2419. 10.1038/s41467-018-04724-5 PMC6010471 29925878

[22] Xu L, Zou C, Zhang S et al. Reshaping the systemic tumor immune environment (STIE) and tumor immune microenvironment (TIME) to enhance immunotherapy efficacy in solid tumors. J Hematol Oncol 2022;15:87. 10.1186/s13045-022-01307-2 PMC9264569 35799264

[23] Baron M, Tagore M, Hunter MV et al. The stress-like cancer cell state is a consistent component of tumorigenesis. Cell Syst 2020;11:536–546.e7. 10.1016/j.cels.2020.08.018 PMC8027961 32910905

[24] Chen W-T, Lu A, Craessaerts K et al. Spatial transcriptomics and In situ sequencing to study Alzheimer’s disease. Cell 2020;182:976–991.e19. 10.1016/j.cell.2020.06.038 32702314

[25] Maniatis S, Äijö T, Vickovic S et al. Spatiotemporal dynamics of molecular pathology in amyotrophic lateral sclerosis. Science 2019;364:89–93. 10.1126/science.aav9776 30948552

[26] Kaufmann M, Schaupp A-L, Sun R et al. Identification of early neurodegenerative pathways in progressive multiple sclerosis. Nat Neurosci 2022;25:944–55. 10.1038/s41593-022-01097-3 35726057

[27] Barmukh R, Garg V, Liu H et al. Spatial omics for accelerating plant research and crop improvement. Trends Biotechnol 2025;43:1904–20. 10.1016/j.tibtech.2025.03.007 40221306

[28] Yin R, Xia K, Xu X. Spatial transcriptomics drives a new era in plant research. The Plant Journal: For Cell and Molecular Biology 2023;116:1571–81. 10.1111/tpj.16437 37651723

[29] Rodriguez PA, Rothballer M, Chowdhury SP et al. Systems biology of plant-microbiome interactions. Mol Plant 2019;12:804–21. 10.1016/j.molp.2019.05.006 31128275

[30] Moses L, Pachter L. Museum of spatial transcriptomics. Nat Methods 2022;19:534–46. 10.1038/s41592-022-01409-2 35273392

[31] Zeng Z, Li Y, Li Y et al. Statistical and machine learning methods for spatially resolved transcriptomics data analysis. Genome Biol 2022;23:83. 10.1186/s13059-022-02653-7 PMC8951701 35337374

[32] Heydari AA, Sindi SS. Deep learning in spatial transcriptomics: learning from the next next-generation sequencing. Biophys Rev 2023;4:011306. 10.1063/5.0091135 PMC10903438 38505815

[33] Luo J, Fu J, Lu Z et al. Deep learning in integrating spatial transcriptomics with other modalities. Brief Bioinform 2024;26:bbae719. 10.1093/bib/bbae719 PMC11725393 39800876

[34] Coleman K, Schroeder A, Li M. Unlocking the power of spatial omics with AI. Nat Methods 2024;21:1378–81. 10.1038/s41592-024-02363-x PMC12023348 39122938

[35] Chen T-Y, You L, Hardillo JAU et al. Spatial Transcriptomic Technologies Cells 2023;12:2042. 10.3390/cells12162042 PMC10453065 37626852

[36] Tian L, Chen F, Macosko EZ. The expanding vistas of spatial transcriptomics. Nat Biotechnol 2023;41:773–82. 10.1038/s41587-022-01448-2 PMC10091579 36192637

[37] Williams CG, Lee HJ, Asatsuma T et al. An introduction to spatial transcriptomics for biomedical research. Genome Med 2022;14:68. 10.1186/s13073-022-01075-1 PMC9238181 35761361

[38] Asp M, Bergenstråhle J, Lundeberg J. Spatially resolved transcriptomes-next generation tools for tissue exploration. BioEssays: News and Reviews in Molecular, Cellular and Developmental Biology 2020;42:e1900221. 10.1002/bies.201900221 32363691

[39] Cheng Y, Dang S, Zhang Y et al. Sequencing-free whole-genome spatial transcriptomics at single-molecule resolution. Cell 2025;188:6953–6970.e12. 10.1016/j.cell.2025.09.006 PMC12662569 41038164

[40] Lubeck E, Coskun AF, Zhiyentayev T et al. Single-cell in situ RNA profiling by sequential hybridization. Nat Methods 2014;11:360–1. 10.1038/nmeth.2892 PMC4085791 24681720

[41] Chen KH, Boettiger AN, Moffitt JR et al. RNA imaging. Spatially resolved, highly multiplexed RNA profiling in single cells. Science 2015;348:aaa6090. 10.1126/science.aaa6090 PMC4662681 25858977

[42] Chen J, Ke R. Spatial analysis toolkits for RNA in situ sequencing. Wiley Interdiscip Rev: RNA 2024;15:e1842. 10.1002/wrna.1842 38605484

[43] Tang X, Chen J, Zhang X et al. Improved in situ sequencing for high-resolution targeted spatial transcriptomic analysis in tissue sections. Journal of genetics and genomics =. Yi Chuan Xue Bao 2023;50:652–60. 10.1016/j.jgg.2023.02.004 36796537

[44] Femino AM, Fay FS, Fogarty K et al. Visualization of single RNA transcripts in situ. Science 1998;280:585–90. 10.1126/science.280.5363.585 9554849

[45] Yue L, Cheung TH. Visualization of RNA transcripts in muscle stem cells using single-molecule fluorescence In situ hybridization. Methods Mol Biol (Clifton, NJ) 2023;2640:445–52. 10.1007/978-1-0716-3036-5_30 36995612

[46] Shah S, Lubeck E, Schwarzkopf M et al. Single-molecule RNA detection at depth by hybridization chain reaction and tissue hydrogel embedding and clearing. Development 2016;143:2862–7. 10.1242/dev.138560 PMC5004914 27342713

[47] Codeluppi S, Borm LE, Zeisel A et al. Spatial organization of the somatosensory cortex revealed by osmFISH. Nat Methods 2018;15:932–5. 10.1038/s41592-018-0175-z 30377364

[48] Shah S, Lubeck E, Zhou W et al. In situ transcription profiling of single cells reveals spatial Organization of Cells in the mouse hippocampus. Neuron 2016;92:342–57. 10.1016/j.neuron.2016.10.001 PMC5087994 27764670

[49] Eng C-HL, Lawson M, Zhu Q et al. Transcriptome-scale super-resolved imaging in tissues by RNA seqFISH. Nature 2019;568:235–9. 10.1038/s41586-019-1049-y PMC6544023 30911168

[50] Rodriques SG, Stickels RR, Goeva A et al. Slide-seq: a scalable technology for measuring genome-wide expression at high spatial resolution. Science 2019;363:1463–7. 10.1126/science.aaw1219 PMC6927209 30923225

[51] Stickels RR, Murray E, Kumar P et al. Highly sensitive spatial transcriptomics at near-cellular resolution with slide-seqV2. Nat Biotechnol 2021;39:313–9. 10.1038/s41587-020-0739-1 PMC8606189 33288904

[52] He S, Bhatt R, Brown C et al. High-plex imaging of RNA and proteins at subcellular resolution in fixed tissue by spatial molecular imaging. Nat Biotechnol 2022;40:1794–806. 10.1038/s41587-022-01483-z 36203011

[53] Merritt CR, Ong GT, Church SE et al. Multiplex digital spatial profiling of proteins and RNA in fixed tissue. Nat Biotechnol 2020;38:586–99. 10.1038/s41587-020-0472-9 32393914

[54] Houston A. Akoya Biosciences PhenoCycler Fusion (Formerly CODEX) User Guide v1. *protocols.io* 2024 [accessed 2026 Feb 10]. https://www.protocols.io/view/akoya-biosciences-phenocycler-fusion-formerly-code-c7fszjne

[55] 10x Genomics Cell Ranger . [accessed 2026 Feb 10]. https://www.10xgenomics.com/

[56] Wang F, Flanagan J, Su N et al. RNAscope. The Journal of Molecular Diagnostics 2012;14:22–9. 10.1016/j.jmoldx.2011.08.002 PMC3338343 22166544

[57] Resolve Biosciences . [accessed 2026 Feb 10]. https://www.resolvebiosciences.com/

[58] Ke R, Mignardi M, Pacureanu A et al. In situ sequencing for RNA analysis in preserved tissue and cells. Nat Methods 2013;10:857–60. 10.1038/nmeth.2563 23852452

[59] Nilsson M, Malmgren H, Samiotaki M et al. Padlock probes: circularizing oligonucleotides for localized DNA detection. Science 1994;265:2085–8. 10.1126/science.7522346 7522346

[60] Magoulopoulou A, Salas SM, Tiklová K et al. Padlock probe-based targeted In situ sequencing: overview of methods and applications. Annu Rev Genomics Hum Genet 2023;24:133–50. 10.1146/annurev-genom-102722-092013 37018847

[61] Lee JH, Daugharthy ER, Scheiman J et al. Fluorescent in situ sequencing (FISSEQ) of RNA for gene expression profiling in intact cells and tissues. Nat Protoc 2015;10:442–58. 10.1038/nprot.2014.191 PMC4327781 25675209

[62] Lee JH, Daugharthy ER, Scheiman J et al. Highly multiplexed subcellular RNA sequencing in situ. Science 2014;343:1360–3. 10.1126/science.1250212 PMC4140943 24578530

[63] Wang X, Allen WE, Wright MA et al. Three-dimensional intact-tissue sequencing of single-cell transcriptional states. Science 2018;361:eaat5691. 10.1126/science.aat5691 PMC6339868 29930089

[64] Alon S, Goodwin DR, Sinha A et al. Expansion sequencing: spatially precise in situ transcriptomics in intact biological systems. Science 2021;371:eaax2656. 10.1126/science.aax2656 PMC7900882 33509999

[65] Chen X, Sun Y-C, Church GM et al. Efficient in situ barcode sequencing using padlock probe-based BaristaSeq. Nucleic Acids Res 2018;46:e22. 10.1093/nar/gkx1206 PMC5829746 29190363

[66] Visium. https://www.10xgenomics.com/platforms/visium

[67] Vickovic S, Eraslan G, Salmén F et al. High-definition spatial transcriptomics for in situ tissue profiling. Nat Methods 2019;16:987–90. 10.1038/s41592-019-0548-y PMC6765407 31501547

[68] Liu Y, Yang M, Deng Y et al. High-spatial-resolution multi-omics sequencing via deterministic barcoding in tissue. Cell 2020;183:1665–1681.e18. 10.1016/j.cell.2020.10.026 PMC7736559 33188776

[69] Chen A, Liao S, Cheng M et al. Spatiotemporal transcriptomic atlas of mouse organogenesis using DNA nanoball-patterned arrays. Cell 2022;185:1777–1792.e21. 10.1016/j.cell.2022.04.003 35512705

[70] Cho C-S, Xi J, Si Y et al. Microscopic examination of spatial transcriptome using Seq-scope. Cell 2021;184:3559–3572.e22. 10.1016/j.cell.2021.05.010 PMC8238917 34115981

[71] Fu X, Sun L, Dong R et al. Polony gels enable amplifiable DNA stamping and spatial transcriptomics of chronic pain. Cell. 2022;185:4621–4633.e17. 10.1016/j.cell.2022.10.021 PMC9691594 36368323

[72] Marx V . Method of the year: spatially resolved transcriptomics. Nat Methods 2021;18:9–14. 10.1038/s41592-020-01033-y 33408395

[73] Quan Y, Zhang H, Wang M et al. Visium spatial transcriptomics reveals intratumor heterogeneity and profiles of Gleason score progression in prostate cancer. iScience 2023;26:108429. 10.1016/j.isci.2023.108429 PMC10698261 38077153

[74] Wirth J, Huber N, Yin K et al. Spatial transcriptomics using multiplexed deterministic barcoding in tissue. Nat Commun 2023;14:1523. 10.1038/s41467-023-37111-w PMC10024691 36934108

[75] Zhao H, Tian G, Hu A. Matrix-Seq: An Adjustable-Resolution Spatial Transcriptomics Via Microfluidic Matrix-Based Barcoding. *bioRxiv* 2022 [accessed 2025 Dec 19]. http://biorxiv.org/lookup/doi/10.1101/2022.08.05.502952

[76] Zhu J, Pang K, Hu B et al. Custom microfluidic chip design enables cost-effective three-dimensional spatiotemporal transcriptomics with a wide field of view. Nat Genet 2024;56:2259–70. 10.1038/s41588-024-01906-4 PMC11525186 39256584

[77] Liu X, Peng T, Xu M et al. Spatial multi-omics: deciphering technological landscape of integration of multi-omics and its applications. J Hematol Oncol 2024;17:72. 10.1186/s13045-024-01596-9 PMC11344930 39182134

[78] Nichterwitz S, Chen G, Aguila Benitez J et al. Laser capture microscopy coupled with smart-seq2 for precise spatial transcriptomic profiling. Nat Commun 2016;7:12139. 10.1038/ncomms12139 PMC4941116 27387371

[79] Chung SH, Shen W. Laser capture microdissection: from its principle to applications in research on neurodegeneration. Neural Regen Res 2015;10:897–8. 10.4103/1673-5374.158346 PMC4498348 26199603

[80] Junker JP, Noël ES, Guryev V et al. Genome-wide RNA tomography in the zebrafish embryo. Cell 2014;159:662–75. 10.1016/j.cell.2014.09.038 25417113

[81] Gurtowski J, Cancio A, Shah H et al. Geoseq: a tool for dissecting deep-sequencing datasets. BMC Bioinformatics 2010;11:506. 10.1186/1471-2105-11-506 PMC2972303 20939882

[82] Boisset J-C, Vivié J, Grün D et al. Mapping the physical network of cellular interactions. Nat Methods 2018;15:547–53. 10.1038/s41592-018-0009-z 29786092

[83] Koch L . Transcriptional profiling of physically interacting cells. Nat Rev Genet 2020;21:275. 10.1038/s41576-020-0229-9 32203278

[84] Manco R, Averbukh I, Porat Z et al. Clump sequencing exposes the spatial expression programs of intestinal secretory cells. Nat Commun 2021;12:3074. 10.1038/s41467-021-23245-2 PMC8144370 34031373

[85] Medaglia C, Giladi A, Stoler-Barak L et al. Spatial reconstruction of immune niches by combining photoactivatable reporters and scRNA-seq. Science 2017;358:1622–6. 10.1126/science.aao4277 PMC7234837 29217582

[86] Lovatt D, Ruble BK, Lee J et al. Transcriptome in vivo analysis (TIVA) of spatially defined single cells in live tissue. Nat Methods 2014;11:190–6. 10.1038/nmeth.2804 PMC3964595 24412976

[87] Honcharuk V, Zainab A, Horimoto Y, Takemoto K, Diez D, Kawaoka S, Vandenbon A. DeepSpaceDB: A Spatial Transcriptomics Atlas for Interactive in-Depth Analysis of Tissues and Tissue Microenvironments. *bioRxiv* 2025 [accessed 2026 Feb 10]. http://biorxiv.org/lookup/doi/10.1101/2025.01.05.631419 10.1093/nar/gkaf1117 PMC12807613 41160880

[88] Li Y, Ding Y, Dennis S et al. SOAR elucidates biological insights and empowers drug discovery through spatial transcriptomics. Science. Advances 2025;11:eadt7450. 10.1126/sciadv.adt7450 PMC13109963 40498826

[89] Zhou W, Su M, Jiang T et al. SORC: An integrated spatial omics resource in cancer. Nucleic Acids Res 2024;52:D1429–37. 10.1093/nar/gkad820 PMC10768140 37811897

[90] Xu Z, Wang W, Yang T et al. STOmicsDB: a comprehensive database for spatial transcriptomics data sharing, analysis and visualization. Nucleic Acids Res 2024;52:D1053–61. 10.1093/nar/gkad933 PMC10767841 37953328

[91] Wang G, Wu S, Xiong Z et al. CROST: a comprehensive repository of spatial transcriptomics. Nucleic Acids Res 2024;52:D882–90. 10.1093/nar/gkad782 PMC10773281 37791883

[92] Fan Z, Luo Y, Lu H et al. SPASCER: spatial transcriptomics annotation at single-cell resolution. Nucleic Acids Res 2023;51:D1138–49. 10.1093/nar/gkac889 PMC9825565 36243975

[93] Zheng Y, Chen Y, Ding X et al. Aquila: a spatial omics database and analysis platform. Nucleic Acids Res 2023;51:D827–34. 10.1093/nar/gkac874 PMC9825501 36243967

[94] Yuan Z, Pan W, Zhao X et al. SODB facilitates comprehensive exploration of spatial omics data. Nat Methods 2023;20:387–99. 10.1038/s41592-023-01773-7 36797409

[95] Fan Z, Chen R, Chen X. SpatialDB: a database for spatially resolved transcriptomes. Nucleic Acids Res 2020;48:D233–7. 10.1093/nar/gkz934 PMC7145543 31713629

[96] Jackson KC, Pachter L. A standard for sharing spatial transcriptomics data. Cell Genom 2023;3:100374. 10.1016/j.xgen.2023.100374 PMC10435375 37601972

[97] Wilkinson MD, Dumontier M, IjJ A et al. The FAIR guiding principles for scientific data management and stewardship. Sci Data 2016;3:160018. 10.1038/sdata.2016.18 PMC4792175 26978244

[98] Luecken MD, Theis FJ. Current best practices in single-cell RNA-seq analysis: a tutorial. Mol Syst Biol 2019;15:e8746. 10.15252/msb.20188746 PMC6582955 31217225

[99] Dries R, Zhu Q, Dong R et al. Giotto: a toolbox for integrative analysis and visualization of spatial expression data. Genome Biol 2021;22:78. 10.1186/s13059-021-02286-2 PMC7938609 33685491

[100] Kleino I, Frolovaitė P, Suomi T et al. Computational solutions for spatial transcriptomics. Comput Struct Biotechnol J 2022;20:4870–84. 10.1016/j.csbj.2022.08.043 PMC9464853 36147664

[101] Palla G, Spitzer H, Klein M et al. Squidpy: a scalable framework for spatial omics analysis. Nat Methods 2022;19:171–8. 10.1038/s41592-021-01358-2 PMC8828470 35102346

[102] Bressan D, Battistoni G, Hannon GJ. The dawn of spatial omics. Science 2023;381:eabq4964. 10.1126/science.abq4964 PMC7614974 37535749

[103] Liu B, Li Y, Zhang L. Analysis and visualization of spatial transcriptomic data. Front Genet 2021;12:785290. 10.3389/fgene.2021.785290 PMC8829434 35154244

[104] Hörl D, Rojas Rusak F, Preusser F et al. BigStitcher: reconstructing high-resolution image datasets of cleared and expanded samples. Nat Methods 2019;16:870–4. 10.1038/s41592-019-0501-0 31384047

[105] Schneider CA, Rasband WS, Eliceiri KW. NIH image to ImageJ: 25 years of image analysis. Nat Methods 2012;9:671–5. 10.1038/nmeth.2089 PMC5554542 22930834

[106] Chiu C-L, Clack N. The napari community. Napari: a python multi-dimensional image viewer platform for the research community. Microsc Microanal 2022;28:1576–7. 10.1017/S1431927622006328

[107] Klein S, Staring M, Murphy K et al. Elastix: a toolbox for intensity-based medical image registration. IEEE Trans Med Imaging 2010;29:196–205. 10.1109/TMI.2009.2035616 19923044

[108] Ma H, Zhang X, Qu Y et al. Vispro improves imaging analysis for Visium spatial transcriptomics. Genome Biol 2025;26:173. 10.1186/s13059-025-03648-w PMC12177973 40533768

[109] Eichenberger BT, Zhan Y, Rempfler M et al. deepBlink: threshold-independent detection and localization of diffraction-limited spots. Nucleic Acids Res 2021;49:7292–7. 10.1093/nar/gkab546 PMC8287908 34197605

[110] Weigert M, Schmidt U. Nuclei instance segmentation and classification in histopathology images with Stardist. In: 2022 IEEE International Symposium on Biomedical Imaging Challenges (ISBIC). Kolkata, India: IEEE; 2022. p. 1–4. https://ieeexplore.ieee.org/document/9854534/. 10.1109/ISBIC56247.2022.9854534

[111] Stringer C, Wang T, Michaelos M et al. Cellpose: a generalist algorithm for cellular segmentation. Nat Methods 2021;18:100–6. 10.1038/s41592-020-01018-x 33318659

[112] Liu Y, Wang T, Duggan B et al. SPCS: a spatial and pattern combined smoothing method for spatial transcriptomic expression. Brief Bioinform 2022;23:bbac116. 10.1093/bib/bbac116 PMC9116229 35380614

[113] Ge Y, Leng J, Tang Z et al. Deep learning-enabled integration of histology and transcriptomics for tissue spatial profile analysis. Research (Wash DC) 2025;8:0568. 10.34133/research.0568 PMC11739434 39830364

[114] Gyllborg D, Langseth CM, Qian X et al. Hybridization-based in situ sequencing (HybISS) for spatially resolved transcriptomics in human and mouse brain tissue. Nucleic Acids Res 2020;48:e112. 10.1093/nar/gkaa792 PMC7641728 32990747

[115] Fang S, Chen B, Zhang Y et al. Computational approaches and challenges in spatial transcriptomics. Genomics Proteomics Bioinformatics 2023;21:24–47. 10.1016/j.gpb.2022.10.001 PMC10372921 36252814

[116] Xi J, Lee JH, Kang HM et al. STtools: a comprehensive software pipeline for ultra-high-resolution spatial transcriptomics data Rattray M, editor. Bioinformatics Advances 2022;2:vbac061. 10.1093/bioadv/vbac061 PMC9590442 36284674

[117] Dobin A, Davis CA, Schlesinger F et al. STAR: Ultrafast universal RNA-seq aligner. Bioinformatics 2013;29:15–21. 10.1093/bioinformatics/bts635 PMC3530905 23104886

[118] Kim D, Langmead B, Salzberg SL. HISAT: a fast spliced aligner with low memory requirements. Nat Methods 2015;12:357–60. 10.1038/nmeth.3317 PMC4655817 25751142

[119] Yue L, Liu F, Hu J et al. A guidebook of spatial transcriptomic technologies, data resources and analysis approaches. Comput Struct Biotechnol J 2023;21:940–55. 10.1016/j.csbj.2023.01.016 PMC10781722 38213887

[120] Lee Y, Lee M, Shin Y et al. Spatial omics in clinical research: a comprehensive review of technologies and guidelines for applications. Int J Mol Sci 2025;26:3949. 10.3390/ijms26093949 PMC12071594 40362187

[121] Zhang Y, Hou Q. Towards a Better Understanding of Batch Effects in Spatial Transcriptomics: Definition and Method Evaluation. *bioRxiv* 2025 [accessed 2026 Aug 11]. http://biorxiv.org/lookup/doi/10.1101/2025.03.12.642755

[122] Fang D, Min W. SpaCross deciphers spatial structures and corrects batch effects in multi-slice spatially resolved transcriptomics. Commun Biol 2025;8:1393. 10.1038/s42003-025-08810-5 PMC12484869 41028333

[123] Korsunsky I, Millard N, Fan J et al. Fast, sensitive and accurate integration of single-cell data with harmony. Nat Methods 2019;16:1289–96. 10.1038/s41592-019-0619-0 PMC6884693 31740819

[124] Zhou H, Panwar P, Guo B et al. Spatial mutual nearest neighbors for spatial transcriptomics data Mathelier a, editor. Bioinformatics 2025;41:btaf403. 10.1093/bioinformatics/btaf403 PMC12317746 40713795

[125] Guo T, Yuan Z, Pan Y et al. SPIRAL: integrating and aligning spatially resolved transcriptomics data across different experiments, conditions, and technologies. Genome Biol 2023;24:241. 10.1186/s13059-023-03078-6 PMC10590036 37864231

[126] Zhong C, Ang KS, Chen J. Interpretable spatially aware dimension reduction of spatial transcriptomics with STAMP. Nat Methods 2024;21:2072–83. 10.1038/s41592-024-02463-8 PMC11541207 39407016

[127] Shang L, Zhou X. Spatially aware dimension reduction for spatial transcriptomics. Nat Commun 2022;13:7203. 10.1038/s41467-022-34879-1 PMC9684472 36418351

[128] Dong K, Zhang S. Deciphering spatial domains from spatially resolved transcriptomics with an adaptive graph attention auto-encoder. Nat Commun 2022;13:1739. 10.1038/s41467-022-29439-6 PMC8976049 35365632

[129] Hu J, Li X, Coleman K et al. SpaGCN: integrating gene expression, spatial location and histology to identify spatial domains and spatially variable genes by graph convolutional network. Nat Methods 2021;18:1342–51. 10.1038/s41592-021-01255-8 34711970

[130] Teng H, Yuan Y, Bar-Joseph Z. Clustering spatial transcriptomics data Kendziorski C, editor. Bioinformatics 2022;38:997–1004. 10.1093/bioinformatics/btab704 PMC8796363 34623423

[131] Kang L, Zhang Q, Qian F et al. Benchmarking computational methods for detecting spatial domains and domain-specific spatially variable genes from spatial transcriptomics data. Nucleic Acids Res 2025;53:gkaf303. 10.1093/nar/gkaf303 PMC12000868 40240000

[132] Traag VA, Waltman L, van Eck NJ. From Louvain to Leiden: guaranteeing well-connected communities. Sci Rep 2019;9:5233. 10.1038/s41598-019-41695-z PMC6435756 30914743

[133] Zhao E, Stone MR, Ren X et al. Spatial transcriptomics at subspot resolution with BayesSpace. Nat Biotechnol 2021;39:1375–84. 10.1038/s41587-021-00935-2 PMC8763026 34083791

[134] Long Y, Ang KS, Li M et al. Spatially informed clustering, integration, and deconvolution of spatial transcriptomics with GraphST. Nat Commun 2023;14:1155. 10.1038/s41467-023-36796-3 PMC9977836 36859400

[135] Pham D, Tan X, Balderson B et al. Robust mapping of spatiotemporal trajectories and cell–cell interactions in healthy and diseased tissues. Nat Commun 2023;14:7739. 10.1038/s41467-023-43120-6 PMC10676408 38007580

[136] Gaspard-Boulinc LC, Gortana L, Walter T et al. Cell-type deconvolution methods for spatial transcriptomics. Nat Rev Genet 2025;26:828–46. 10.1038/s41576-025-00845-y 40369312

[137] Li H, Zhou J, Li Z et al. A comprehensive benchmarking with practical guidelines for cellular deconvolution of spatial transcriptomics. Nat Commun 2023;14:1548. 10.1038/s41467-023-37168-7 PMC10027878 36941264

[138] Zhang Y, Lin X, Yao Z et al. Deconvolution algorithms for inference of the cell-type composition of the spatial transcriptome. Comput Struct Biotechnol J 2023;21:176–84. 10.1016/j.csbj.2022.12.001 PMC9755226 36544473

[139] Lopez R, Li B, Keren-Shaul H et al. DestVI identifies continuums of cell types in spatial transcriptomics data. Nat Biotechnol 2022;40:1360–9. 10.1038/s41587-022-01272-8 PMC9756396 35449415

[140] Kleshchevnikov V, Shmatko A, Dann E et al. Cell2location maps fine-grained cell types in spatial transcriptomics. Nat Biotechnol 2022;40:661–71. 10.1038/s41587-021-01139-4 35027729

[141] Ma Y, Zhou X. Spatially informed cell-type deconvolution for spatial transcriptomics. Nat Biotechnol 2022;40:1349–59. 10.1038/s41587-022-01273-7 PMC9464662 35501392

[142] Cable DM, Murray E, Zou LS et al. Robust decomposition of cell type mixtures in spatial transcriptomics. Nat Biotechnol 2022;40:517–26. 10.1038/s41587-021-00830-w PMC8606190 33603203

[143] Cheng J, Jin X, Smyth GK et al. Benchmarking cell type annotation methods for 10x xenium spatial transcriptomics data. BMC Bioinformatics 2025;26:22. 10.1186/s12859-025-06044-0 PMC11744978 39833693

[144] Jin K, Zhang Z, Zhang K et al. Bering: joint cell segmentation and annotation for spatial transcriptomics with transferred graph embeddings. Nat Commun 2025;16:6618. 10.1038/s41467-025-60898-9 PMC12274425 40681510

[145] Li Z, M.Patel Z, Song D et al. Systematic benchmarking of computational methods to identify spatially variable genes. Genome Biol 2025;26:285. 10.1186/s13059-025-03731-2 PMC12445034 40968359

[146] Das Adhikari S, Yang J, Wang J et al. Recent advances in spatially variable gene detection in spatial transcriptomics. Comput Struct Biotechnol J 2024;23:883–91. 10.1016/j.csbj.2024.01.016 PMC10869304 38370977

[147] Chen C, Kim HJ, Yang P. Evaluating spatially variable gene detection methods for spatial transcriptomics data. Genome Biol 2024;25:18. 10.1186/s13059-023-03145-y PMC10789051 38225676

[148] Zhu J, Sun S, Zhou X. SPARK-X: non-parametric modeling enables scalable and robust detection of spatial expression patterns for large spatial transcriptomic studies. Genome Biol 2021;22:184. 10.1186/s13059-021-02404-0 PMC8218388 34154649

[149] Moran PAP . The interpretation of statistical maps. Journal of the Royal Statistical Society Series B: Statistical Methodology 1948;10:243–51. 10.1111/j.2517-6161.1948.tb00012.x

[150] Zhang C, Dong K, Aihara K et al. STAMarker: determining spatial domain-specific variable genes with saliency maps in deep learning. Nucleic Acids Res 2023;51:e103. 10.1093/nar/gkad801 PMC10639070 37811885

[151] Acharyya S, Zhou X, Baladandayuthapani V. SpaceX: gene co-expression network estimation for spatial transcriptomics. Bioinformatics 2022;38:5033–41. 10.1093/bioinformatics/btac645 PMC9665869 36179087

[152] van Santvoort M, Eduati F. An algorithmic perspective on deciphering cell-cell interactions with spatial omics data. Brief Bioinform 2025;26:bbaf236. 10.1093/bib/bbaf236 PMC12103901 40413871

[153] Holdener C, Vlaminck D, I. Smoothie: efficient inference of spatial Co-expression networks from denoised spatial transcriptomics data. bioRxiv: The Preprint Server for Biology 2025:2025.02.26.640406. 10.1101/2025.02.26.640406 PMC13031863 41888598

[154] Li Z, Wang T, Liu P et al. SpatialDM for rapid identification of spatially co-expressed ligand-receptor and revealing cell-cell communication patterns. Nat Commun 2023;14:3995. 10.1038/s41467-023-39608-w PMC10325966 37414760

[155] Jin S, Guerrero-Juarez CF, Zhang L et al. Inference and analysis of cell-cell communication using CellChat. Nat Commun 2021;12:1088. 10.1038/s41467-021-21246-9 PMC7889871 33597522

[156] Browaeys R, Saelens W, Saeys Y. NicheNet: Modeling intercellular communication by linking ligands to target genes. Nat Methods 2020;17:159–62. 10.1038/s41592-019-0667-5 31819264

[157] Liu J, Manabe H, Qian W, Wang Y, Gu Y, Yan Chu AK, Gadhvi G, Song Y, Ono N, Welch JD. CytoSignal Detects Locations and Dynamics of Ligand-Receptor Signaling at Cellular Resolution from Spatial Transcriptomic Data. *bioRxiv* 2024 [accessed 2025 Dec 19]. http://biorxiv.org/lookup/doi/10.1101/2024.03.08.584153 10.1038/s41588-026-02624-9 PMC13263138 42271087

[158] Wang H, Zhao J, Nie Q et al. Dissecting spatiotemporal structures in spatial transcriptomics via diffusion-based adversarial learning. Research (Wash DC) 2024;7:0390. 10.34133/research.0390 PMC11134684 38812530

[159] Yu Y, Nie W, Zhang Q et al. Inferring causal trajectories from spatial transcriptomics using CASCAT. Nucleic Acids Res 2025;53:gkaf791. 10.1093/nar/gkaf791 PMC12362255 40829806

[160] Ren J, Zhou H, Zeng H et al. Spatiotemporally resolved transcriptomics reveals the subcellular RNA kinetic landscape. Nat Methods 2023;20:695–705. 10.1038/s41592-023-01829-8 PMC10172111 37038000

[161] Wang S, Lin S, Yang C. The dawn of spatiotemporal transcriptomics. Biomedical Analysis 2024;1:154–61. 10.1016/j.bioana.2024.06.002

[162] Shen X, Zuo L, Ye Z et al. Inferring cell trajectories of spatial transcriptomics via optimal transport analysis. Cell Syst 2025;16:101194. 10.1016/j.cels.2025.101194 39904341

[163] Long W, Liu T, Xue L et al. spVelo: RNA velocity inference for multi-batch spatial transcriptomics data. Genome Biol 2025;26:239. 10.1186/s13059-025-03701-8 PMC12337411 40790237

[164] Aivazidis A, Memi F, Kleshchevnikov V et al. Cell2fate infers RNA velocity modules to improve cell fate prediction. Nat Methods 2025;22:698–707. 10.1038/s41592-025-02608-3 PMC11978503 40032996

[165] Lee H, Sun H, Cao X, Karaahmet B, Li Z, Ulrich-Klein H, Taga M, Wang G, De Jager PL, Bennett DA et al. Mapping Disease Critical Spatially Variable Gene Programs by Integrating Spatial Transcriptomics with Human Genetics. *bioRxiv* 2025 [accessed 2025 Dec 19]. http://biorxiv.org/lookup/doi/10.1101/2025.09.24.678397

[166] Tran T, Wee F, Joseph CR et al. An integrated approach for analyzing spatially resolved multi-omics datasets from the same tissue section. Front Mol Biosci 2025;12:1614288. 10.3389/fmolb.2025.1614288 PMC12304548 40735471

[167] Liu Y, Wang C, Wang Z et al. High-parameter spatial multi-omics through histology-anchored integration. Nat Methods 2026;23:373–86. 10.1038/s41592-025-02926-6 41407925

[168] Huang J, Fu X, Zhang Z et al. A graph self-supervised residual learning framework for domain identification and data integration of spatial transcriptomics. Commun Biol 2024;7:1123. 10.1038/s42003-024-06814-1 PMC11393357 39266614

[169] Birk S, Bonafonte-Pardàs I, Feriz AM et al. Quantitative characterization of cell niches in spatially resolved omics data. Nat Genet 2025;57:897–909. 10.1038/s41588-025-02120-6 PMC11985353 40102688

[170] Singhal V, Chou N, Lee J et al. BANKSY unifies cell typing and tissue domain segmentation for scalable spatial omics data analysis. Nat Genet 2024;56:431–41. 10.1038/s41588-024-01664-3 PMC10937399 38413725

[171] Argelaguet R, Arnol D, Bredikhin D et al. MOFA+: a statistical framework for comprehensive integration of multi-modal single-cell data. Genome Biol 2020;21:111. 10.1186/s13059-020-02015-1 PMC7212577 32393329

[172] Schott M, León-Periñán D, Splendiani E et al. Open-ST: high-resolution spatial transcriptomics in 3D. Cell 2024;187:3953–3972.e26. 10.1016/j.cell.2024.05.055 38917789

[173] Zhao H, Yin X, Wang S et al. So3D: a comprehensive three-dimensional spatial omics resource for decoding tissue architecture in physiology and disease. Nucleic Acids Res 2025;8:gkaf998. 10.1093/nar/gkaf998 PMC12808542 41062448

[174] Sui X, Lo JA, Luo S et al. Scalable spatial single-cell transcriptomics and translatomics in 3D thick tissue blocks. Nat Methods 2025;22:2574–84. 10.1038/s41592-025-02867-0 41286200

[175] Túrós D, Gladiseva L, Botos M, He C, Barut GT, Veiga IB, Ebert N, Bonnin A, Chanfon A, Grau-Roma L et al. Deep Learning-Based 3D Spatial Transcriptomics with X-Pression. *bioRxiv* 2025 [accessed 2025 Dec 19]. http://biorxiv.org/lookup/doi/10.1101/2025.03.21.644627

[176] Li B, Bao F, Sun Y, Wang T, Li F, Li H, Dai Q, Deng Y *.* Three-Dimensional Spatial Transcriptomics at Isotropic Resolution Enabled by Generative Deep Learning. *bioRxiv* 2025 [accessed 2025 Dec 19]. http://biorxiv.org/lookup/doi/10.1101/2025.08.15.670472

[177] Niu J, Fang D, Chen J et al. SpaBatch: deep learning-based Cross-slice integration and 3D spatial domain identification in spatial transcriptomics. Advanced Science (Weinheim, Baden-Wurttemberg, Germany) 2025;12:e09090. 10.1002/advs.202509090 PMC12667491 40953305

[178] Qing J, Wu J, Li Y et al. SCNT: An R package for data analysis and visualization of single-cell and spatial transcriptomics. BMC Bioinformatics 2025;26:184. 10.1186/s12859-025-06209-x PMC12273005 40681987

[179] Ospina OE, Manjarres-Betancur R, Gonzalez-Calderon G et al. spatialGE: a user-friendly web application to democratize spatial transcriptomics analysis. bioRxiv 2024. [accessed 2026 Feb 10]. 10.1101/2024.06.27.601050 PMC11873723 39636739

[180] Keller MS, Gold I, McCallum C et al. Vitessce: integrative visualization of multimodal and spatially resolved single-cell data. Nat Methods 2025;22:63–7. 10.1038/s41592-024-02436-x PMC11725496 39333268

[181] Pielawski N, Andersson A, Avenel C et al. TissUUmaps 3: improvements in interactive visualization, exploration, and quality assessment of large-scale spatial omics data. Heliyon 2023;9:e15306. 10.1016/j.heliyon.2023.e15306 PMC10149187 37131430

[182] Mohanty C, Prasad A, Cheng L et al. SpatialView: An interactive web application for visualization of multiple samples in spatial transcriptomics experiments. Bioinformatics 2024;40:btae117. 10.1093/bioinformatics/btae117 PMC10957517 38444087

[183] Chen X, Ran Q, Tang J et al. Benchmarking algorithms for spatially variable gene identification in spatial transcriptomics Mathelier a, editor. Bioinformatics 2025;41:btaf131. 10.1093/bioinformatics/btaf131 PMC12036962 40139667

[184] Yan Y, Gu T, Sun C et al. Benchmarking alignment methods for spatial transcriptomics data. Nature Computational Science 2026;6:524–41. 10.1038/s43588-026-00977-z 41933187

[185] Dong K, Gao Y, Zou Q et al. Benchmarking multi-slice integration and downstream applications in spatial transcriptomics data analysis. Genome Biol 2025;26:318. 10.1186/s13059-025-03796-z PMC12477787 41024097

[186] Tchatchoua Ngassam B, Niu H, Pang S et al. Applications of AI to single-cell and spatial transcriptomics: current state-of-the-art and challenges. Front Bioinform 2026;5:1715821. 10.3389/fbinf.2025.1715821 PMC12886477 41676376

[187] Cao J, Li C, Cui Z et al. Spatial transcriptomics: a powerful tool in disease understanding and drug discovery. Theranostics 2024;14:2946–68. 10.7150/thno.95908 PMC11103497 38773973

[188] Duan H, Cheng T, Cheng H. Spatially resolved transcriptomics: advances and applications. Blood Sci 2023;5:1–14. 10.1097/BS9.0000000000000141 PMC9891446 36742187

[189] Jain S, Eadon MT. Spatial transcriptomics in health and disease. Nat Rev Nephrol 2024;20:659–71. 10.1038/s41581-024-00841-1 PMC11392631 38719971

[190] Cilento MA, Sweeney CJ, Butler LM. Spatial transcriptomics in cancer research and potential clinical impact: a narrative review. J Cancer Res Clin Oncol 2024;150:296. 10.1007/s00432-024-05816-0 PMC11162383 38850363

[191] Jin Y, Zuo Y, Li G et al. Advances in spatial transcriptomics and its applications in cancer research. Mol Cancer 2024;23:129. 10.1186/s12943-024-02040-9 PMC11188176 38902727

[192] See J-E, Barlow S, Arjumand W et al. Spatial omics: applications and utility in profiling the tumor microenvironment. Cancer Metastasis Rev 2025;44:87. 10.1007/s10555-025-10304-z PMC12672822 41331191

[193] Croizer H, Mhaidly R, Kieffer Y et al. Deciphering the spatial landscape and plasticity of immunosuppressive fibroblasts in breast cancer. Nat Commun 2024;15:2806. 10.1038/s41467-024-47068-z PMC10984943 38561380

[194] Xie L, Kong H, Yu J et al. Spatial transcriptomics reveals heterogeneity of histological subtypes between lepidic and acinar lung adenocarcinoma. Clin Transl Med 2024;14:e1573. 10.1002/ctm2.1573 PMC10844893 38318637

[195] Arora R, Cao C, Kumar M et al. Spatial transcriptomics reveals distinct and conserved tumor core and edge architectures that predict survival and targeted therapy response. Nat Commun 2023;14:5029. 10.1038/s41467-023-40271-4 PMC10439131 37596273

[196] Jing S-Y, Wang H-Q, Lin P et al. Quantifying and interpreting biologically meaningful spatial signatures within tumor microenvironments. NPJ Precis Oncol 2025;9:68. 10.1038/s41698-025-00857-1 PMC11897387 40069556

[197] Zhang Y, Gong S, Liu X. Spatial transcriptomics: a new frontier in accurate localization of breast cancer diagnosis and treatment. Front Immunol 2024;15:1483595. 10.3389/fimmu.2024.1483595 PMC11493667 39439806

[198] Maciejewski K, Czerwinska P. Scoping review: methods and applications of spatial transcriptomics in tumor research. Cancers 2024;16:3100. 10.3390/cancers16173100 PMC11394603 39272958

[199] Yu Q, Jiang M, Wu L. Spatial transcriptomics technology in cancer research. Front Oncol 2022;12:1019111. 10.3389/fonc.2022.1019111 PMC9606570 36313703

[200] Li C, Xu C, Guan R et al. Spatial transcriptomics reveal tumor microenvironment and SLCO2A1 correlated with tumor suppression in hypopharyngeal squamous cell carcinoma. Int Immunopharmacol 2024;142:113243. 10.1016/j.intimp.2024.113243 39340989

[201] Huang B, Chen Y, Yuan S. Application of spatial transcriptomics in digestive system Tumors. Biomolecules 2024;15:21. 10.3390/biom15010021 PMC11761220 39858416

[202] Dagogo-Jack I, Shaw AT. Tumour heterogeneity and resistance to cancer therapies. Nat Rev Clin Oncol 2018;15:81–94. 10.1038/nrclinonc.2017.166 29115304

[203] Bao Z, Wang Y, Wang Q et al. Intratumor heterogeneity, microenvironment, and mechanisms of drug resistance in glioma recurrence and evolution. Front Med 2021;15:551–61. 10.1007/s11684-020-0760-2 33893983

[204] Jung N, Kim T-K. Spatial transcriptomics in neuroscience. Exp Mol Med 2023;55:2105–15. 10.1038/s12276-023-01093-y PMC10618223 37779145

[205] Zhang L, Xiong Z, Xiao M. A review of the application of spatial transcriptomics in neuroscience. Interdiscip Sci 2024;16:243–60. 10.1007/s12539-024-00603-4 38374297

[206] Ya D, Zhang Y, Cui Q et al. Application of spatial transcriptome technologies to neurological diseases. Front Cell Dev Biol 2023;11:1142923. 10.3389/fcell.2023.1142923 PMC10020196 36936681

[207] Wang M, Zhuo L, Ma W et al. AllenDigger, a tool for spatial expression data visualization, spatial heterogeneity delineation, and single-cell registration based on the Allen brain atlas. J Phys Chem A 2023;127:2864–72. 10.1021/acs.jpca.3c00145 PMC10068737 36926884

[208] Wang Q, Zhu H, Deng L et al. Spatial transcriptomics: biotechnologies, computational tools, and neuroscience applications. Small Methods 2025;9:e2401107. 10.1002/smtd.202401107 39760243

[209] Wang Y, Liu B, Zhao G et al. Spatial transcriptomics: technologies, applications and experimental considerations. Genomics 2023;115:110671. 10.1016/j.ygeno.2023.110671 PMC10571167 37353093

[210] Lee CYC, McCaffrey J, McGovern D et al. Profiling immune cell tissue niches in the spatial -omics era. J Allergy Clin Immunol 2025;155:663–77. 10.1016/j.jaci.2024.11.001 39522655

[211] De Giovanni M, Inverso D, Iannacone M. From histology to high-resolution mapping: the rise of spatial omics in immunology. Eur J Immunol 2025;55:e70073. 10.1002/eji.70073 PMC12501406 41053988

[212] Wan Y, El Kholtei J, Jenie I, Colomer-Rosell M, Liu J, Acedo JN, Du LY, Codina-Tobias M, Wang M, Sawh A et al. Whole-Embryo Spatial Transcriptomics at Subcellular Resolution from Gastrulation to Organogenesis. *bioRxiv* 2024 [accessed 2026 Feb 10]. http://biorxiv.org/lookup/doi/10.1101/2024.08.27.609868 10.1126/science.adt3439 41818362

[213] Single-cell and spatial transcriptomics during human organogenesis. Nat Cell Biol 2023;25:522–3. 10.1038/s41556-023-01113-z PMC10032247 36949166

[214] Tam PPL, Loebel DAF. Gene function in mouse embryogenesis: get set for gastrulation. Nat Rev Genet 2007;8:368–81. 10.1038/nrg2084 17387317

[215] Sampath Kumar A, Tian L, Bolondi A et al. Spatiotemporal transcriptomic maps of whole mouse embryos at the onset of organogenesis. Nat Genet 2023;55:1176–85. 10.1038/s41588-023-01435-6 PMC10335937 37414952

[216] Choe K, Pak U, Pang Y et al. Advances and challenges in spatial transcriptomics for developmental biology. Biomolecules 2023;13:156. 10.3390/biom13010156 PMC9855858 36671541

[217] Holdener C, Jiang S, Sutherland DM et al. Spatial transcriptomics to study virus-host interactions. Annu Rev Virol 2025;12:379–99. 10.1146/annurev-virology-092623-104926 PMC12683572 40523107

[218] Chen Q, Yuan Y, Cai F et al. Single-nucleus and spatial transcriptomics revealing host response differences triggered by mutated virus in severe dengue. Viruses 2024;16:1779. 10.3390/v16111779 PMC11599075 39599894

[219] Cross AR, de Andrea CE, Villalba-Esparza M et al. Spatial transcriptomic characterization of COVID-19 pneumonitis identifies immune circuits related to tissue injury. JCI Insight 2023;8:e157837. 10.1172/jci.insight.157837 PMC9977306 36472908

[220] Kasmani MY, Topchyan P, Brown AK et al. A spatial sequencing atlas of age-induced changes in the lung during influenza infection. Nat Commun 2023;14:6597. 10.1038/s41467-023-42021-y PMC10584893 37852965

[221] Chen S, Zhang Y, Ashuo A et al. Combination of spatial transcriptomics analysis and retrospective study reveals liver infection of SARS-COV-2 is associated with clinical outcomes of COVID-19. EBioMedicine 2025;111:105517. 10.1016/j.ebiom.2024.105517 PMC11732063 39709771

[222] Zhang T, Li Y, Pan L et al. Brain-wide alterations revealed by spatial transcriptomics and proteomics in COVID-19 infection. Nat Aging 2024;4:1598–618. 10.1038/s43587-024-00730-z PMC11867587 39543407

[223] Prebensen C, Lefol Y, Myhre PL et al. Longitudinal whole blood transcriptomic analysis characterizes neutrophil activation and interferon signaling in moderate and severe COVID-19. Sci Rep 2023;13:10368. 10.1038/s41598-023-37606-y PMC10293211 37365222

[224] Guan Q, Eshetu A, Ho Y-C. Roadmap for spatial transcriptomics of HIV in tissues. Curr Opin HIV AIDS 2025;20:502–11. 10.1097/COH.0000000000000961 PMC13336902 40626838

[225] Qiu X, Zhong P, Yue L et al. Spatial transcriptomic sequencing reveals immune microenvironment features of mycobacterium tuberculosis granulomas in lung and omentum. Theranostics 2024;14:6185–201. 10.7150/thno.99038 PMC11488093 39431015

[226] Mantri M, Hinchman MM, McKellar DW et al. Spatiotemporal transcriptomics reveals pathogenesis of viral myocarditis. Nature Cardiovascular Research 2022;1:946–60. 10.1038/s44161-022-00138-1 PMC10035375 36970396

[227] Krull D, Haynes P, Kesarwani A et al. A best practices framework for spatial biology studies in drug discovery and development: enabling successful cohort studies using digital spatial profiling. J Histotechnol 2025;48:7–26. 10.1080/01478885.2024.2391683 39225147

[228] Chu X, Tian Y, Lv C. Decoding the spatiotemporal heterogeneity of tumor-associated macrophages. Mol Cancer 2024;23:150. 10.1186/s12943-024-02064-1 PMC11282869 39068459

[229] He B, Bergenstråhle L, Stenbeck L et al. Integrating spatial gene expression and breast tumour morphology via deep learning. Nat Biomed Eng 2020;4:827–34. 10.1038/s41551-020-0578-x 32572199

[230] Deng P, Huang J, He W et al. Opportunities and challenges in the application of spatiotemporal transcriptomics in plant research. Front Plant Sci 2025;16:1684057. 10.3389/fpls.2025.1684057 PMC12568615 41169728

[231] Giacomello S . A new era for plant science: spatial single-cell transcriptomics. Curr Opin Plant Biol 2021;60:102041. 10.1016/j.pbi.2021.102041 33915520

[232] Sang Q, Kong F. Applications for single-cell and spatial transcriptomics in plant research. New Crops 2024;1:100025. 10.1016/j.ncrops.2024.100025

[233] Lv Z, Jiang S, Kong S et al. Advances in single-cell transcriptome sequencing and spatial transcriptome sequencing in plants. Plants 2024;13:1679. 10.3390/plants13121679 PMC11207393 38931111

[234] Li Y, Stanojevic S, Garmire LX. Emerging artificial intelligence applications in spatial transcriptomics analysis. Comput Struct Biotechnol J 2022;20:2895–908. 10.1016/j.csbj.2022.05.056 PMC9201012 35765645

[235] Schmauch B, Herpin L, Olivier A et al. A deep learning-based multiscale integration of spatial omics with tumor morphology. Nat Commun 2025;16:11674. 10.1038/s41467-025-66691-y PMC12748640 41310346

[236] Huang Z, Bianchi F, Yuksekgonul M et al. A visual-language foundation model for pathology image analysis using medical twitter. Nat Med 2023;29:2307–16. 10.1038/s41591-023-02504-3 37592105

[237] Zhang S, Xu Y, Usuyama N, Xu H, Bagga J, Tinn R, Preston S, Rao R, Wei M, Valluri N et al. BiomedCLIP: A Multimodal Biomedical Foundation Model Pretrained from Fifteen Million Scientific Image-Text Pairs. *arXiv* 2023 [accessed 2026 Feb 10]. https://arxiv.org/abs/2303.00915. 10.48550/ARXIV.2303.00915

[238] Radford A, Kim JW, Hallacy C, Ramesh A, Goh G, Agarwal S, Sastry G, Askell A, Mishkin P, Clark J et al. Learning Transferable Visual Models from Natural Language Supervision. *arXiv* 2021 [accessed 2026 Aug 5]. https://arxiv.org/abs/2103.00020. 10.48550/ARXIV.2103.00020

[239] Lu MY, Chen B, Williamson DFK et al. A visual-language foundation model for computational pathology. Nat Med 2024;30:863–74. 10.1038/s41591-024-02856-4 PMC11384335 38504017

[240] Jang J, Kyung D, Kim SH et al. Significantly improving zero-shot X-ray pathology classification via fine-tuning pre-trained image-text encoders. Sci Rep 2024;14:23199. 10.1038/s41598-024-73695-z PMC11455863 39369048

[241] Sun S, Zhu J, Zhou X. Statistical analysis of spatial expression patterns for spatially resolved transcriptomic studies. Nat Methods 2020;17:193–200. 10.1038/s41592-019-0701-7 PMC7233129 31988518

[242] Khan T, Farley CM, Wilson JJ et al. Tackling the complexity of spatial transcriptomics data interpretation with large language models. bioRxiv 2024. [accessed 2026 Feb 10]. 10.1101/2024.11.28.625773

[243] Huang CH . QuST-LLM: Integrating Large Language Models for Comprehensive Spatial Transcriptomics Analysis. *arXiv* 2024 [accessed 2026 Feb 10]. https://arxiv.org/abs/2406.14307. 10.48550/ARXIV.2406.14307

[244] Li L, Dong L, Zhang H et al. spaLLM: enhancing spatial domain analysis in multi-omics data through large language model integration. Brief Bioinform 2025;26:bbaf304. 10.1093/bib/bbaf304 PMC12224616 40608008

[245] Wei H, Luo X, Yu H, Liang J, Yang L, Lin L, Popa A, Yan X. Identifying cellular niches in spatial transcriptomics: An investigation into the capabilities of large language models. In: Editors Che W, Nabende J, Shutova E, Pilehvar MT (eds.), Proceedings of the 63rd Annual Meeting of the Association for Computational Linguistics (Volume 1: Long Papers). Vienna, Austria: Association for Computational Linguistics; 2025. p. 9275–89. https://aclanthology.org/2025.acl-long.455. 10.18653/v1/2025.acl-long.455

[246] Liu S, Tang J, Ma J, Liang S. TissueNarrator: Generative Modeling of Spatial Transcriptomics with Large Language Models. *bioRxiv* 2025 [accessed 2026 Feb 10]. http://biorxiv.org/lookup/doi/10.1101/2025.11.24.690325

[247] Choi H, Shin H, Lee D, Lee D. Generative Single-Cell Transcriptomics Via Large Language Models. *bioRxiv* 2026 [accessed 2026 Feb 10]. 10.64898/2026.01.12.699186

[248] Liu T, Huang T, Ding T et al. Leveraging multi-modal foundation models for analysing spatial multi-omic and histopathology data. Nat Biomed Eng 2026;10:1714–31. 10.1038/s41551-025-01602-6 41644824

[249] Li S, Xiao T, Lan Y et al. Artificial intelligence revolution in transcriptomics: from single cells to spatial atlases. Adv Sci 2026;13:e18949. 10.1002/advs.202518949 PMC12850002 41387122

[250] Molla Desta G, Birhanu AG. Advancements in single-cell RNA sequencing and spatial transcriptomics: transforming biomedical research. Acta Biochim Pol 2025;72:13922. 10.3389/abp.2025.13922 PMC11835515 39980637

[251] Khan M, Arslanturk S, Draghici S. A comprehensive review of spatial transcriptomics data alignment and integration. Nucleic Acids Res 2025;53:gkaf536. 10.1093/nar/gkaf536 PMC12199153 40568931

[252] Chen C, Ge Y, Lu L. Opportunities and challenges in the application of single-cell and spatial transcriptomics in plants. Front Plant Sci 2023;14:1185377. 10.3389/fpls.2023.1185377 PMC10453814 37636094

[253] Smith KD, Prince DK, MacDonald JW et al. Challenges and opportunities for the clinical translation of spatial transcriptomics technologies. Glomerular Dis 2024;4:49–63. 10.1159/000538344 PMC11006413 38600956

[254] Wang H, Cheng P, Wang J et al. Advances in spatial transcriptomics and its application in the musculoskeletal system. Bone Res 2025;13:54. 10.1038/s41413-025-00429-w PMC12084619 40379648

[255] Zhou R, Yang G, Zhang Y et al. Spatial transcriptomics in development and disease. Mol Biomed 2023;4:32. 10.1186/s43556-023-00144-0 PMC10560656 37806992

[256] Li B, Tang Z, Budhkar A et al. SpaIM: single-cell spatial transcriptomics imputation via style transfer. Nat Commun 2025;16:7861. 10.1038/s41467-025-63185-9 PMC12375071 40849313

[257] Li B, Zhang Y, Wang Q et al. Gene expression prediction from histology images via hypergraph neural networks. Brief Bioinform 2024;25:bbae500. 10.1093/bib/bbae500 PMC11472757 39401144

[258] Ge S, Sun S, Xu H et al. Deep learning in single-cell and spatial transcriptomics data analysis: advances and challenges from a data science perspective. Brief Bioinform 2025;26:bbaf136. 10.1093/bib/bbaf136 PMC11970898 40185158

[259] Manogaran S, Ramadoss R, Selvam SP et al. Artificial intelligence-driven spatial transcriptomics in OSCC: mapping the tumor microenvironment and personalizing therapy. Journal of oral biology and craniofacial. Research 2025;15:1862–73. 10.1016/j.jobcr.2025.10.015 PMC12639440 41282271

[260] Almagro-Pérez C, Song AH, Weishaupt L, Kim A, Jaume G, Williamson DFK, Hemker K, Lu MY, Singh K, Chen B et al. AI-Driven 3D Spatial Transcriptomics*. arXiv* 2025 [accessed 2026 Feb 10]. https://arxiv.org/abs/2502.17761. 10.48550/ARXIV.2502.17761

[261] Chelebian E, Avenel C, Wählby C. Combining spatial transcriptomics with tissue morphology. Nat Commun 2025;16:4452. 10.1038/s41467-025-58989-8 PMC12075478 40360467

[262] Laury AR, Zheng S, Aho N et al. Opening the Black box: spatial transcriptomics and the relevance of artificial intelligence-detected prognostic regions in high-grade serous carcinoma. Modern Pathology: An Official Journal of the United States and Canadian Academy of Pathology, Inc 2024;37:100508. 10.1016/j.modpat.2024.100508 38704029

